# A Novel tRNA Half mt‐5’‐tiRNA‐Tyr Promotes Colorectal Cancer Proliferation via Inducing HARS2 Succinylation

**DOI:** 10.1002/advs.77042

**Published:** 2026-08-21

**Authors:** Xinliang Gu, Danping Zhu, Xinwei Liu, Shangshang Hu, Xincheng Yang, Junjie Nie, Tao Xu, Yuqin Pan, Mu Xu, Huiling Sun, Shukui Wang

**Affiliations:** ^1^ General Clinical Research Center Nanjing First Hospital Nanjing Medical University Nanjing Jiangsu China; ^2^ School of Pharmacy China Pharmaceutical University Nanjing Jiangsu China; ^3^ Department of Laboratory Medicine Nanjing First Hospital Nanjing Medical University Nanjing Jiangsu China; ^4^ Jiangsu Collaborative Innovation Center on Cancer Personalized Medicine Nanjing Medical University Nanjing Jiangsu China; ^5^ Nanjing Medical Key Laboratory of Laboratory Diagnostics Nanjing Jiangsu China

**Keywords:** colorectal cancer, HARS2, mt‐5’‐tiRNA‐Tyr, succinylation, tRNA‐derived small RNAs

## Abstract

Transfer RNA‐derived small RNAs (tsRNAs) are a novel class of small non‐coding RNAs abundant in the bloodstream of cancer patients and involved in various physiological and pathological processes. However, the regulatory mechanisms and clinical significance of tsRNAs in colorectal cancer (CRC) remain unclear. In this study, PANDORA‐seq was used as an exploratory screen of CRC tissues, paired adjacent tissues, and CRC plasma samples, followed by validation in larger independent cohorts. Quantitative real‐time PCR quantified mt‐5’‐tiRNA‐Tyr levels in tissues, plasma, and cells. Functional assays assessed the role of mt‐5’‐tiRNA‐Tyr in CRC. Mechanistic studies utilized RNA pull‐down, mass spectrometry, RNA immunoprecipitation, western blotting, and Co‐immunoprecipitation. Results revealed that mt‐5’‐tiRNA‐Tyr was significantly upregulated in CRC plasma and tissues. In vitro and in vivo studies demonstrated its oncogenic potential. Mechanistic assays showed that mt‐5’‐tiRNA‐Tyr was enriched with HARS2 in RNA pull‐down/RIP assays and associated with reduced HARS2‐mt‐tRNA‐His association, increased HARS2 K91 succinylation, impaired mitochondrial protein translation, and mitochondrial damage. Moreover, succinate accumulation creates a positive feedback loop that enhances CRC proliferation. This study identifies a novel oncogenic tsRNA and uncovers a mechanism by which mt‐5’‐tiRNA‐Tyr promotes CRC proliferation through HARS2 succinylation, supporting further evaluation of plasma mt‐5’‐tiRNA‐Tyr as a candidate circulating biomarker for CRC.

## Graphical abstract

A mitochondria‐derived tRNA half, mt‐5’‐tiRNA‐Tyr, is upregulated in colorectal cancer tissues and plasma and promotes tumor proliferation. It binds HARS2 and promotes CPT1A‐associated HARS2 K91 succinylation, disrupts HARS2–mt‐tRNA‐His association, impairs mitochondrial translation, enhances succinate‐linked metabolic reprogramming, and shows promise as a circulating biomarker for colorectal cancer.

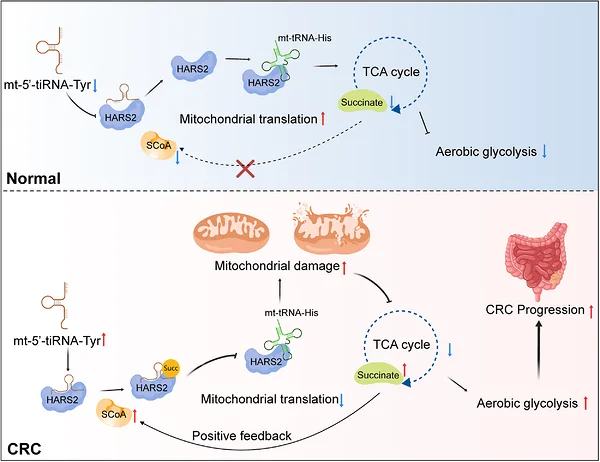

## 1 Introduction

Colorectal cancer (CRC) ranks as the third leading cause of both morbidity and mortality among malignancies, significantly affecting public health and quality of life [1]. Early screening and diagnosis are vital for reducing CRC mortality, but current methods have limitations. Colonoscopy, considered the gold standard, is costly, complex, and invasive, which hinders its widespread use [2]. Non‐invasive tests, such as fecal occult blood and immunochemical tests, are less expensive but lack sensitivity and specificity [3, 4]. Fecal DNA methylation testing is accurate but expensive, time‐consuming, and prone to instability [5]. Plasma tumor markers like carcinoembryonic antigen (CEA), carbohydrate antigen 199 (CA19‐9), and carbohydrate antigen 72–4 (CA72‐4) have limited sensitivity and specificity, especially for early diagnosis [6]. Consequently, the identification of novel non‐invasive biomarkers with enhanced sensitivity and specificity is crucial for the screening and early detection of CRC.

The rapid advancement of high‐throughput sequencing technologies has underscored transfer RNA‐derived small RNAs (tsRNAs) as a pivotal area of research [7, 8]. These small non‐coding RNAs, generated through the cleavage of mature or precursor tRNA molecules, are classified into tRNA halves (tiRNAs) and tRNA‐derived fragments (tRFs) based on their length and specific cleavage sites [9]. Notably, tsRNAs can be detected non‐invasively in blood with high sensitivity and specificity, rendering them valuable as biomarkers [10, 11]. For example, Wu et al. demonstrated that the levels of 5'‐tRF‐GlyGCC were significantly elevated in the plasma of patients with CRC, exhibiting strong diagnostic potential, particularly when used in conjunction with carcinoembryonic antigen CEA and CA19‐9 [12]. The stability of tsRNAs in peripheral blood, even after repeated cycles of freezing and thawing, coupled with their ease of detection, positions them as promising biomarkers. Consequently, the identification of novel tsRNAs has become a significant area of interest within the research community.

In this study, we identified an mt‐5’‐tiRNA‐Tyr that is highly expressed in CRC tissues and plasma using PANDORA‐seq. In vitro and in vivo experiments demonstrated that elevated levels of mt‐5’‐tiRNA‐Tyr facilitate the proliferation of CRC. Mechanistically, mt‐5’‐tiRNA‐Tyr overexpression is associated with increased HARS2 succinylation at the K91 site and reduced HARS2‐mt‐tRNA‐His association. This altered HARS2‐RNA association is accompanied by impaired mitochondrial protein translation, a shift in CRC cell energy metabolism from the tricarboxylic acid cycle to aerobic glycolysis, and increased CRC proliferation. The resulting succinate buildup further enhances HARS2 succinylation, creating a feedback loop that intensifies CRC proliferation. Additionally, we demonstrate that mt‐5’‐tiRNA‐Tyr holds potential as a plasma biomarker for CRC.

## 2 Results

### 2.1 Expression Profile of tsRNAs in CRC Tissues and Plasma

To identify candidate tsRNAs suitable for further liquid biopsy validation in CRC, we conducted PANDORA‐seq as an exploratory screening analysis using CRC tissues, paired adjacent tissues, and plasma samples from three CRC patients (Figure 1A). Considering the limited sample size of this initial sequencing cohort, candidate tsRNAs identified from this screening step were subsequently subjected to validation in larger independent tissue and plasma cohorts. Principal component analysis (PCA) demonstrated a distinct expression profile between the CRC tissues (C group) and the adjacent tissues (N group) (Figure 1B). Correlation coefficient analysis revealed a significant relationship between the two groups (Figure 1C). Subsequently, we categorized tsRNAs into four groups according to the reference [13]: tsRNA‐5, tsRNA‐3, tsRNA‐CCA, and tsRNA‐ other. Notably, tsRNA‐5 accounted for approximately 30% of the total tsRNAs in both the C and N groups. Furthermore, the types of tRNAs that give rise to tsRNAs are illustrated in Figure 1E. Heatmaps and scatter plots were employed to visualize the expression levels and distribution patterns of tsRNAs within the C and N groups (Figure 1F,G). Additionally, correlation coefficient analysis indicated a significant correlation among the three plasma samples (Figure 1H). In the study, tsRNA‐5 accounted for approximately 6% of the total tsRNAs, a proportion significantly different from that observed in tissue samples (Figure 1I). Subsequent analysis of tsRNA sequencing data identified 1631 tsRNAs present in CRC tissues, adjacent tissues, and plasma samples (Figure 1J). From these, 127 tsRNAs were identified as highly expressed, based on a Fold change > 2 and a *p*‐value < 0.05. We then intersected the 1000 tsRNAs with the highest expression levels in plasma with those highly expressed in tissues. This analysis revealed that 20 tsRNAs were significantly overexpressed in both tissue and plasma samples (Figure 1K). Among these 20 overlapping tsRNAs, six candidates were selected for subsequent validation according to predefined criteria. First, the candidate tsRNA had to show consistently higher expression in CRC tissues than in paired adjacent tissues across all three sequenced tissue pairs. Second, the sequence length had to be at least 18 nt, because fragments shorter than 18 nt are less favorable for stable and specific qRT‐PCR detection based on our experimental experience. Third, the candidate tsRNA had to show relatively high abundance in CRC plasma sequencing data. Based on these criteria, six tsRNAs were selected for further validation by qRT‐PCR in plasma samples. To further determine whether mt‐5’‐tiRNA‐Tyr represented a discrete small RNA species in the sequencing data, we analyzed the read length distribution of reads mapped to the mt‐tRNA‐Tyr locus. In pooled CRC tissues, reads mapped to this locus showed a clear enrichment at 31 nt, and 31 nt reads accounted for 36.4% of mt‐tRNA‐Tyr locus reads. In pooled adjacent tissues, 31 nt reads accounted for 17.6% of mt‐tRNA‐Tyr locus reads, with a more heterogeneous length distribution (Figure S1A). These results support the enrichment of a discrete 31 nt mt‐tRNA‐Tyr derived fragment in CRC tissues. We further analyzed the exact sequences of reads mapped to the mt‐tRNA‐Tyr locus. The exact 31 nt sequence corresponding to mt‐5’‐tiRNA‐Tyr was GGTAAAATGGCTGAGTGAAGCATTGGACTGT. This exact fragment represented the most abundant exact fragment mapped to the mt‐tRNA‐Tyr locus, accounting for 27.0% of all pooled mt‐tRNA‐Tyr locus reads (Figure S1B). In addition, this exact mt‐5’‐tiRNA‐Tyr sequence accounted for 99.4% of all 31 nt reads mapped to the mt‐tRNA‐Tyr locus (Figure S1C), indicating that the 31 nt reads were dominated by a single exact sequence. We next quantified the abundance of the exact 31 nt mt‐5’‐tiRNA‐Tyr fragment. In pooled CRC tissues, this fragment accounted for 36.2% of mt‐tRNA‐Tyr locus reads and 3787 reads per million total tsRNA reads. In pooled adjacent tissues, it accounted for 17.5% of mt‐tRNA‐Tyr locus reads and 1296 reads per million total tsRNA reads. Across all pooled tissue samples, the exact 31 nt mt‐5’‐tiRNA‐Tyr fragment accounted for 27.0% of mt‐tRNA‐Tyr locus reads and 2349 reads per million total tsRNA reads (Figure S1C,D). qRT‐PCR validation in plasma samples showed that mt‐5’‐tiRNA‐Tyr, derived from mt‐tRNA‐Tyr‐GTA, was significantly elevated in CRC plasma compared with healthy donor plasma (Figure 1L), prompting further investigation into its potential biological role and clinical relevance.

**FIGURE 1:**
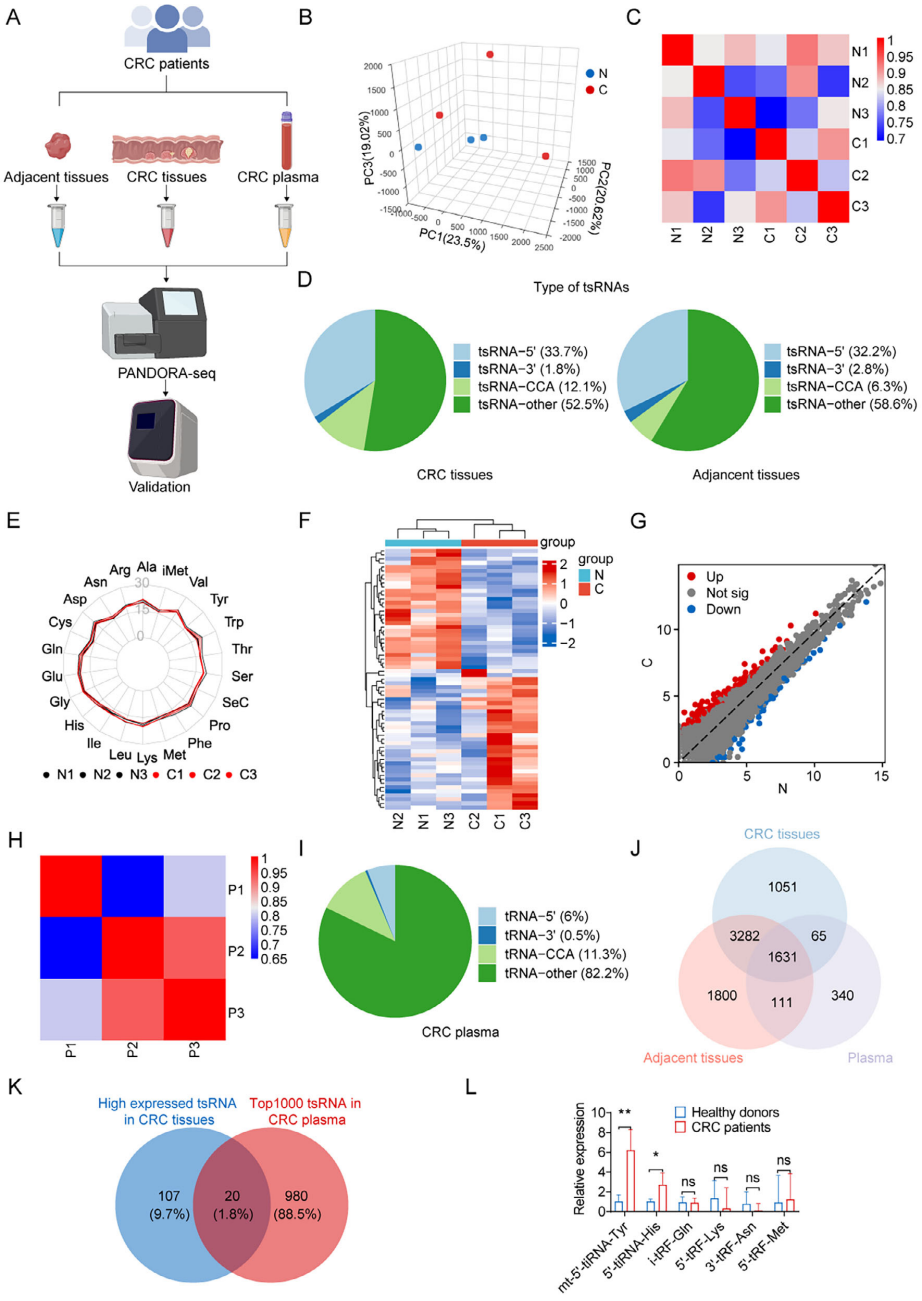
Expression profile of tsRNAs in CRC tissues and plasma. (A) The workflow of PANDORA‐seq and validation of the study. (B) The PCA of tsRNAs expression in N and C samples. (C) The correlation coefficient analysis of tsRNAs expression in N and C samples. (D) Distributions of different types of tsRNAs in N and C samples. (E) Origin of tsRNAs in N and C samples. (F) Heatmap of tsRNAs expression in N and C samples. (G) Scatter plot of tsRNAs expression in N and C samples. (H) The correlation coefficient analysis of tsRNAs expression in CRC plasma samples. (I) Distributions of different types of tsRNAs expression in CRC plasma samples. (J) Venn diagram of tsRNAs expression in N, C, and CRC plasma samples. (K) Venn diagram of 20 tsRNAs, which were significantly highly expressed in CRC tissues and had high expression levels in CRC plasma. (L) Expression level of the top 6 tsRNAs in 19 CRC plasma. All values are means ± SD, and significance was determined by the two‐tailed Student's *t*‐test.

### 2.2 mt‐5’‐tiRNA‐Tyr is up‐Regulated in CRC

The UCSC database indicated that mt‐5’‐tiRNA‐Tyr is localized on chromosome chrMT at positions 5861–5891, with a length of 31 nt (Figure 2A). MINTbase V2.0 identified it as a 5’‐half derived from mt‐tRNA‐Tyr‐GTA (Figure 2B), and the predicted cleavage site is illustrated in Figure 2C. FISH analysis showed that the mt‐5’‐tiRNA‐Tyr probe signal largely overlapped with TOM20‐positive mitochondrial regions in SW480 cells (Figure 2D). To further evaluate the mitochondrial enrichment of mt‐5’‐tiRNA‐Tyr, we isolated mitochondrial and cytosolic fractions from SW480 cells and quantified mt‐5’‐tiRNA‐Tyr using the stem‐loop qRT‐PCR assay used throughout this study. mt‐5’‐tiRNA‐Tyr was predominantly enriched in the mitochondrial fraction, similar to the mitochondrial transcript MTATP8, whereas GAPDH was mainly detected in the cytosolic fraction (Figure 2E).

**FIGURE 2:**
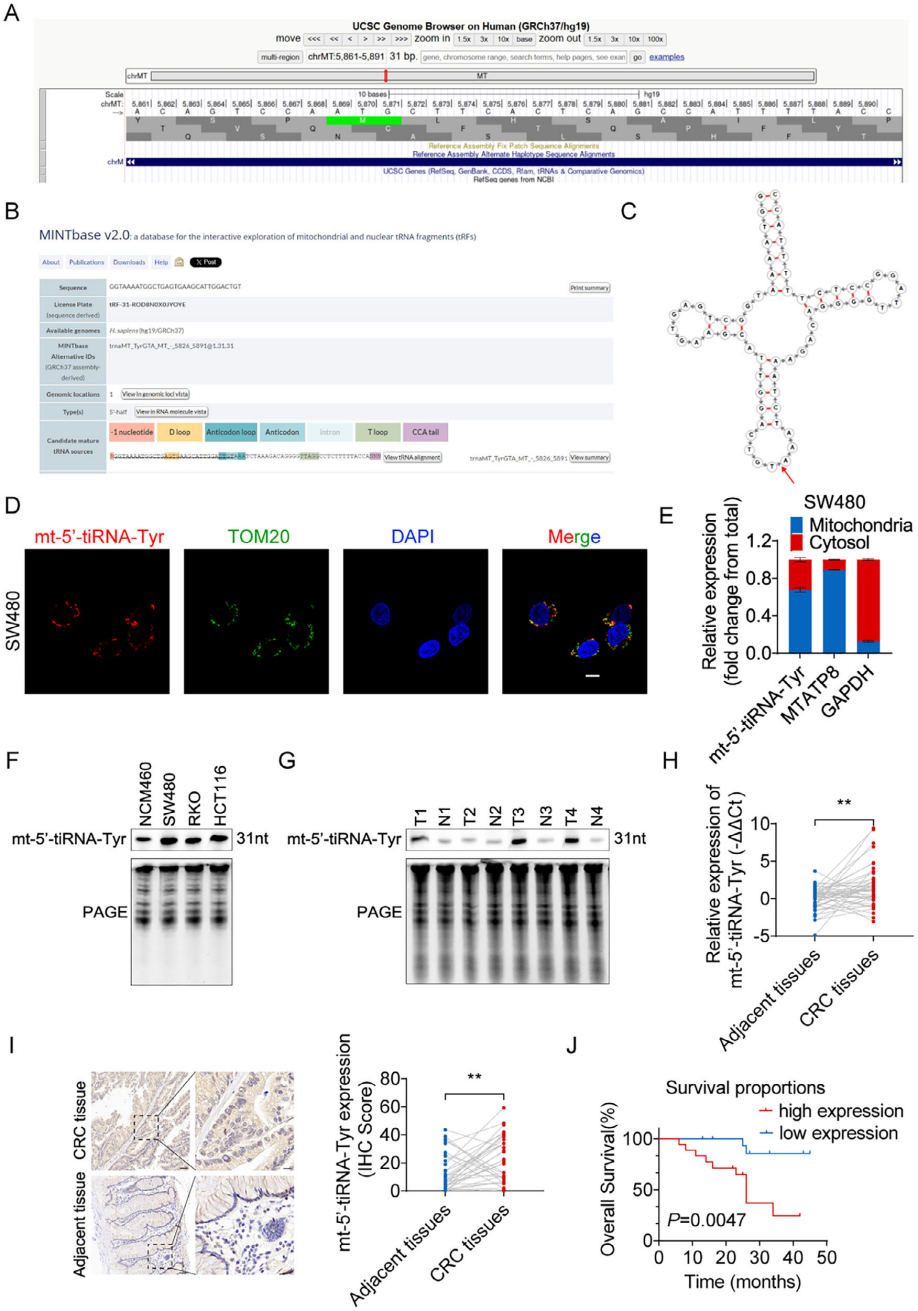
mt‐5’‐tiRNA‐Tyr is upregulated in CRC tissues. (A) UCSC Genome Browser view showing the genomic location of mt‐5’‐tiRNA‐Tyr at chrMT:5861–5891. (B) Basic information of mt‐5’‐tiRNA‐Tyr from MINTbase v2.0. (C) Predicted cleavage site of mt‐5’‐tiRNA‐Tyr in mature mt‐tRNA‐Tyr. (D) Representative FISH images showing mt‐5’‐tiRNA‐Tyr probe signal (red), TOM20 (green), and DAPI (blue) in SW480 cells. Scale bar, 10 µm. (E) qRT‐PCR analysis of mt‐5’‐tiRNA‐Tyr in mitochondrial and cytosolic fractions from SW480 cells. MTATP8 and GAPDH were used as mitochondrial and cytosolic controls, respectively. (F) Northern blot detection of mt‐5’‐tiRNA‐Tyr in NCM460, SW480, RKO, and HCT116 cells. PAGE staining was used as a loading control. (G) Northern blot detection of mt‐5’‐tiRNA‐Tyr in paired CRC tissues and adjacent tissues. PAGE staining was used as a loading control. (H) qRT‐PCR analysis of mt‐5’‐tiRNA‐Tyr expression in 48 paired CRC tissues and adjacent tissues. ^**^ *p* < 0.01. (I) ISH analysis of mt‐5’‐tiRNA‐Tyr probe signal in CRC tissues and adjacent tissues. Scale bar, left: 50 µm; right: 12.5 µm. ^**^ *p* < 0.01. (J) Kaplan–Meier analysis of the association between mt‐5’‐tiRNA‐Tyr expression and overall survival in CRC patients. All values are means ± SD unless otherwise indicated, and significance was determined by the paired two‐tailed Wilcoxon test (H, I) or log‐rank (Mantel–Cox) test (J).

To directly validate the endogenous size and discreteness of mt‐5’‐tiRNA‐Tyr, we performed Northern blot analysis using total RNA from CRC cell lines and tissue samples. A clear and discrete band of approximately 31 nt was detected in CRC cell lines and paired tissue samples, supporting the presence of an endogenous mt‐5’‐tiRNA‐Tyr species of the expected size (Figure 2F,G).

Using the stem‐loop qRT‐PCR assay specifically designed for mt‐5’‐tiRNA‐Tyr, we confirmed that mt‐5’‐tiRNA‐Tyr expression was significantly elevated in CRC tissues compared with adjacent tissues, consistent with the sequencing results (Figure 2H). In addition, agarose gel electrophoresis showed a single qRT‐PCR amplicon consistent with the expected 72‐bp product predicted from the stem‐loop qRT‐PCR design. The qRT‐PCR product was then cloned and subjected to Sanger sequencing, which confirmed that the cloned amplicon contained the mt‐5’‐tiRNA‐Tyr‐derived sequence (Figure S1E,F). To explore the potential clinicopathological significance of mt‐5’‐tiRNA‐Tyr expression in CRC, we designed and synthesized a probe targeting mt‐5’‐tiRNA‐Tyr and performed ISH analysis using CRC tissue microarrays. Consistent with the qRT‐PCR results, the ISH signal was higher in CRC tissues than in adjacent tissues (Figure 2I). We interpreted this probe‐based result as supportive evidence for tissue enrichment. Moreover, higher mt‐5’‐tiRNA‐Tyr expression was associated with poorer overall survival in CRC patients (Figure 2J).

### 2.3 mt‐5’‐tiRNA‐Tyr is Upregulated via HIF‐1α/ANG Axis

Tumor growth creates a hypoxic environment, and in CRC, tsRNAs are produced by angiogenin cleavage under such conditions [14]. To understand how mt‐5’‐tiRNA‐Tyr is produced in CRC, we cultured CRC cells in an anoxic incubator for 24–48 h. After 24 h, SW480 and RKO cell lines showed a significant rise in hypoxia‐inducible factor 1‐alpha (HIF‐1α) protein levels (Figure S2A). The levels of mt‐5’‐tiRNA‐Tyr and ANG protein increased with extended hypoxic exposure, linking mt‐5’‐tiRNA‐Tyr production to hypoxia (Figure S2B,C). Using siRNAs to knock down ANG under hypoxia significantly reduced ANG protein and mt‐5’‐tiRNA‐Tyr expression, highlighting ANG's critical role in mt‐5’‐tiRNA‐Tyr generation (Figure S2D,E). Additionally, HIF‐1α is a key transcription factor regulating many genes [15]. We utilized the JASPAR database to predict HIF‐1α binding to the ANG promoter, and subsequently created a plasmid containing the ANG promoter and a luciferase reporter gene. Luciferase assays showed that HIF‐1α overexpression increased luciferase activity via the ANG promoter (Figure S2F,G). Thus, mt‐5’‐tiRNA‐Tyr upregulation is due to HIF‐1α activating ANG transcription under hypoxia in CRC cells.

### 2.4 mt‐5’‐tiRNA‐Tyr Promotes Proliferation of CRC In Vitro and In Vivo

To elucidate the role of mt‐5’‐tiRNA‐Tyr in CRC, we initially observed a significant upregulation of mt‐5’‐tiRNA‐Tyr expression in SW480 and RKO cell lines compared to the NCM460 cell line (Figure 3A). We then transfected SW480 and RKO cells with mt‐5’‐tiRNA‐Tyr inhibitor or mimics and evaluated mt‐5’‐tiRNA‐Tyr levels in both whole cell lysates and isolated mitochondrial fractions by qRT‐PCR. The inhibitor reduced mt‐5’‐tiRNA‐Tyr levels in both whole cell lysates and mitochondrial fractions, whereas the mimics increased mt‐5’‐tiRNA‐Tyr levels in both fractions (Figure 3B,C). These results confirmed that the transfected inhibitor and mimics effectively modulated mt‐5’‐tiRNA‐Tyr levels in the mitochondrial fraction, supporting the use of these reagents for subsequent mitochondrial mechanism related assays. In vitro CCK‐8 and colony formation assays demonstrated that mt‐5’‐tiRNA‐Tyr inhibition reduced CRC cell proliferation, whereas mt‐5’‐tiRNA‐Tyr overexpression enhanced CRC cell proliferation (Figure 3D–G). Before establishing the CDX model, we validated the stable mt‐5’‐tiRNA‐Tyr down SW480 cells by qRT‐PCR. The lentiviral down construct significantly reduced mt‐5’‐tiRNA‐Tyr expression, whereas the parental mt‐tRNA‐Tyr was not significantly altered (Figure 3H). To further assess whether mt‐5’‐tiRNA‐Tyr exerts similar stimulatory effects on CRC in vivo, we established a cell‐derived xenograft (CDX) model (Figure 3I). The mt‐5’‐tiRNA‐Tyr down group showed reduced tumor growth and tumor weight compared with the negative control (NC) group (Figure 3J–L). No obvious difference in body weight was observed between the NC and mt‐5’‐tiRNA‐Tyr down groups (Figure 3M). Furthermore, mt‐5’‐tiRNA‐Tyr inhibition was associated with decreased Ki67 staining and increased Cleaved caspase‐3 staining in tumor tissues (Figure 3N). Collectively, these results suggest that mt‐5’‐tiRNA‐Tyr may facilitate CRC proliferation both in vitro and in vivo.

**FIGURE 3:**
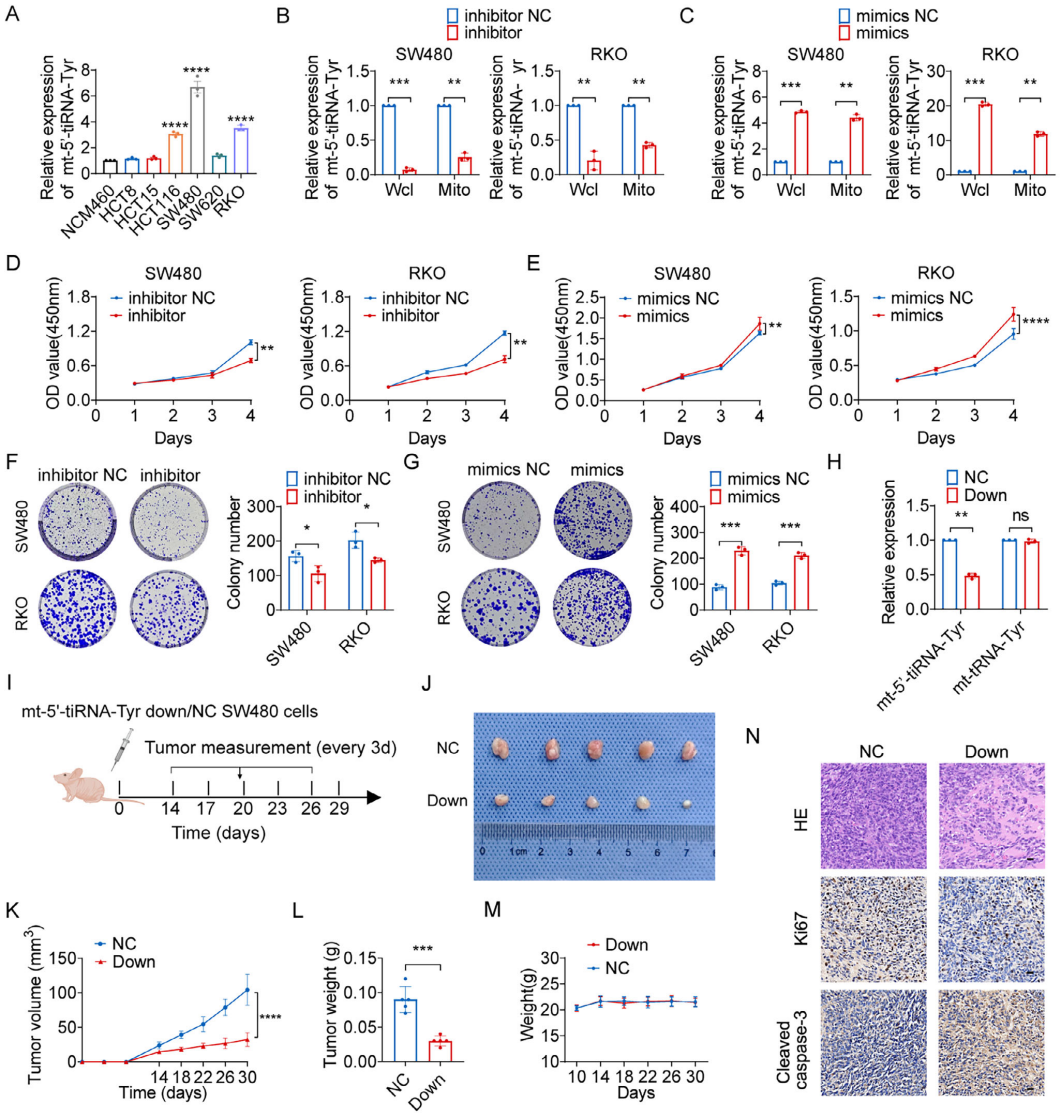
mt‐5’‐tiRNA‐Tyr promotes proliferation of CRC in vitro and in vivo. (A) qRT‐PCR analysis of mt‐5’‐tiRNA‐Tyr expression in CRC cell lines and NCM460 cells (*n* = 3). ^****^ *p* < 0.0001. (B, C) qRT‐PCR analysis of mt‐5’‐tiRNA‐Tyr expression in whole‐cell lysates (WCL) and mitochondrial fractions (Mito) after transfection with mt‐5’‐tiRNA‐Tyr inhibitor (B) or mimics (C) in SW480 and RKO cells (*n* = 3). ^***^ *p* < 0.001, ^**^ *p* < 0.01. (D, E) CCK‐8 assays were performed to evaluate cell proliferation after transfection with mt‐5’‐tiRNA‐Tyr inhibitor (D) or mimics (E) (*n* = 3). ^****^ *p* < 0.0001, ^**^ *p* < 0.01. (F, G) Colony formation assays were performed to evaluate cell proliferation after transfection with mt‐5’‐tiRNA‐Tyr inhibitor (F) or mimics (G) (*n* = 3). ^***^ *p* < 0.001, ^*^ *p* < 0.05. (H) qRT‐PCR analysis of mt‐5’‐tiRNA‐Tyr and parental mt‐tRNA‐Tyr levels in stable NC and mt‐5’‐tiRNA‐Tyr down SW480 cells (*n* = 3). ^**^ *p* < 0.01; ns, not significant. (I) Schematic diagram of the CDX model of CRC. (J–L) Tumor growth in the mt‐5’‐tiRNA‐Tyr down group was suppressed, as shown by representative tumor images (J), tumor volume (K), and tumor weight (L), compared with the NC group (*n* = 5). ^****^ *p* < 0.0001, ^***^ *p* < 0.001. (M) Body weight of mice in the NC and mt‐5’‐tiRNA‐Tyr down groups (*n* = 5). (N) HE staining and IHC analysis of Ki67 and Cleaved caspase‐3 in xenograft tumors. Scale bar, 20 µm. All values are means ± SD, and significance was determined by one‐way ANOVA followed by Tukey's test (A), two‐tailed Student's *t*‐test (B, C, F–H, L), or two‐way ANOVA followed by Tukey's test (D, E, K).

### 2.5 mt‐5’‐tiRNA‐Tyr Associates With HARS2

To further investigate the underlying mechanisms by which mt‐5’‐tiRNA‐Tyr influences the proliferation of CRC, we initially inhibited mt‐5’‐tiRNA‐Tyr in SW480 cells and conducted mRNA sequencing. The analysis of mRNA sequencing data revealed that the transcriptomic profiles of cells in the inhibitor group differed significantly from those in the inhibitor NC group (Figure S3A and Figure 4A,B). Gene Ontology (GO) and Kyoto Encyclopedia of Genes and Genomes (KEGG) analyses indicated that the differentially expressed genes were significantly enriched in pathways related to oxidative phosphorylation and mitochondrial protein‐containing complexes (Figure S3B,C). Subsequently, we employed RNA pull‐down and mass spectrometry assays to identify proteins associated with mt‐5’‐tiRNA‐Tyr. The RNA pull‐down assay was performed using biotin‐labeled mt‐5’‐tiRNA‐Tyr (Figure 4C). Among the proteins identified, HARS2 emerged as the most significant, based on protein abundance rankings (Figure 4D). Furthermore, peptides corresponding to HARS2 were detected, as illustrated in Figure 4E. To corroborate the mass spectrometry results, we performed western blotting analysis, which revealed that the mt‐5’‐tiRNA‐Tyr sense strand effectively pulled down a greater amount of HARS2 protein compared to the antisense strand (Figure 4F). Additionally, RIP assays further detected enrichment of mt‐5’‐tiRNA‐Tyr in HARS2 immunoprecipitates (Figure 4G). To map the HARS2 region associated with mt‐5’‐tiRNA‐Tyr enrichment, we constructed vectors encoding Flag‐tagged full‐length HARS2, as well as truncated variants (1–405aa) and del (62–390aa) (Figure 4H). Each vector was independently transfected into 293T cells, followed by RNA pull‐down assays using an mt‐5’‐tiRNA‐Tyr probe. The results indicated that the HGTP_anticodon‐containing region was required for mt‐5’‐tiRNA‐Tyr enrichment in the pull‐down assay (Figure 4I). RIP assays further confirmed that mt‐5’‐tiRNA‐Tyr was preferentially enriched with the HGTP_anticodon‐containing region, while other domains were not involved (Figure 4J). Subsequently, we utilized the HDOCK server to predict putative contact sites of mt‐5’‐tiRNA‐Tyr on HARS2. A mutant probe was generated by altering these bases (Figure 4K,L), and the findings demonstrated that HARS2 enrichment was largely abolished with the mutated probe (Figure 4M), suggesting that the association between mt‐5’‐tiRNA‐Tyr and HARS2 is dependent on these specific bases.

**FIGURE 4:**
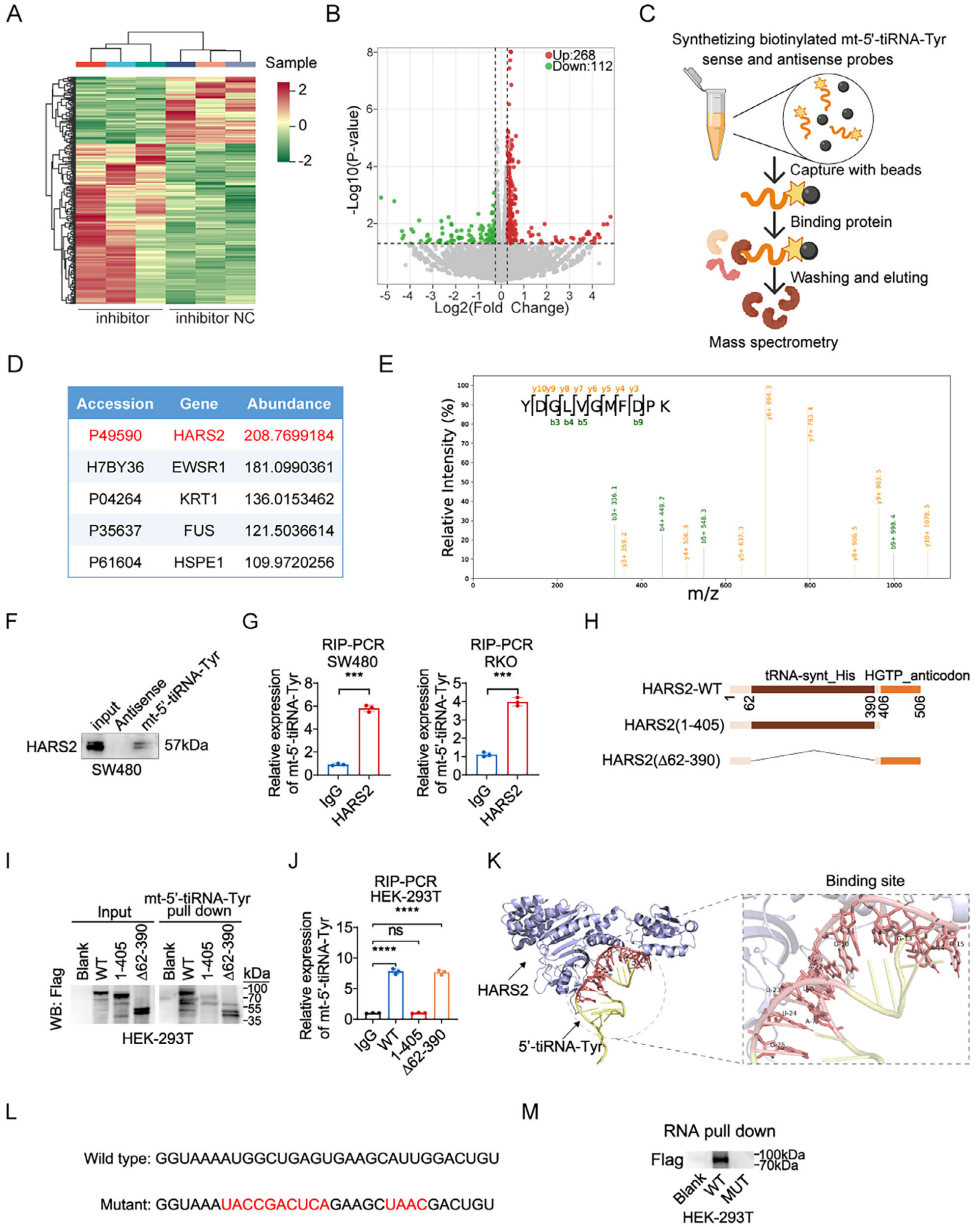
mt‐5’‐tiRNA‐Tyr associates with HARS2. (A) Cluster heat map of the DEGs between mt‐5’‐tiRNA‐Tyr inhibitor and the negative control. (B) Volcano plot of the DEGs between mt‐5’‐tiRNA‐Tyr inhibitor and the negative control. (C) Schematic illustration of extracting endogenous mt‐5’‐tiRNA‐Tyr. (D) The top 5 proteins with the highest abundance identified by mass spectrometry were shown, and HARS2 was identified as a candidate mt‐5’‐tiRNA‐Tyr‐associated protein. (E) The specific peptides of HARS2 identified by mass spectrometry. (F) Independent RNA pulldown and western blotting assays showed HARS2 enrichment in the mt‐5’‐tiRNA‐Tyr sense‐strand pulldown compared with the antisense control in SW480 cells. (G) RIP assay was performed to detect enrichment of mt‐5’‐tiRNA‐Tyr in HARS2 immunoprecipitates from SW480 and RKO cells. ^***^ *p* < 0.001. (H) Schematic diagram shows the Flag‐tagged HARS2 protein domain structure. (I) Western blotting shows Flag‐tagged full‐length (WT) HARS2 and its truncated forms enriched by mt‐5’‐tiRNA‐Tyr. (J) RIP assays with an antibody against Flag showed that WT and Δ62‐390, but not 1–405, showed enrichment of mt‐5’‐tiRNA‐Tyr. ^****^ *p* < 0.0001. (K) Predicted structural model of the putative HARS2‐mt‐5’‐tiRNA‐Tyr complex. (L, M) RNA pulldown assay shows HARS2 enrichment by mutant and wild‐type mt‐5’‐tiRNA‐Tyr probes. All values are means ± SD, and significance was determined by the two‐tailed Student's *t*‐test (G) or one‐way ANOVA followed by Tukey's test (J).

### 2.6 mt‐5’‐tiRNA‐Tyr Increases HARS2 Protein Stability

The analysis of the TCGA dataset indicated that HARS2 expression was significantly elevated in CRC tissues compared to adjacent non‐cancerous tissues (Figure S4A). IHC assays demonstrated that HARS2 protein levels were elevated in CRC tissues compared to adjacent tissues (Figure S4B). To explore the influence of mt‐5’‐tiRNA‐Tyr on HARS2 expression, we transfected SW480 and RKO cell lines with inhibitor and mimics of mt‐5’‐tiRNA‐Tyr, subsequently evaluating the expression levels of HARS2. However, no significant alterations were detected in HARS2 mRNA levels (Figure S4C). Additionally, IHC assays indicated that HARS2 protein levels were decreased in CRC tissues with downregulated mt‐5’‐tiRNA‐Tyr compared to the NC groups (Figure S4D). Notably, the inhibition of mt‐5’‐tiRNA‐Tyr resulted in a reduction of HARS2 protein levels. In contrast, the overexpression of mt‐5’‐tiRNA‐Tyr led to an increase in HARS2 protein levels (Figure S4E). The aforementioned results led us to hypothesize that mt‐5’‐tiRNA‐Tyr may influence HARS2 by modulating its protein stability rather than affecting its transcription. qRT‐PCR results indicated that HARS2 expression was higher in CRC cells (Figure S4F). Then, we established stable SW480 cell lines with HARS2 overexpression and knockdown. Among the knockdown constructs, sh‐HARS2‐2 exhibited the most significant reduction in HARS2 expression; therefore, it was selected for further investigation (Figure S4G,H). Then, we treated SW480 cells overexpressing mt‐5’‐tiRNA‐Tyr with the protein synthesis inhibitor cycloheximide (CHX) and subsequently collected cell lysates for western blotting analysis. As demonstrated in Figure S4I, compared with the NC group, the half‐life of the HARS2 protein in the mt‐5’‐tiRNA‐Tyr overexpression group was significantly prolonged (Figure S4I). These findings suggest that mt‐5’‐tiRNA‐Tyr may regulate HARS2 protein stability by reducing its degradation.

### 2.7 mt‐5’‐tiRNA‐Tyr Increased the Succinylation Level of HARS2 at Lys91

Post‐translational modifications (PTMs) serve as a fundamental mechanism for regulating protein function and are crucial in modulating binding protein expression and function by tsRNAs [16]. To investigate the mechanism by which mt‐5’‐tiRNA‐Tyr regulates HARS2, we assessed the levels of various PTMs on HARS2 following the overexpression of mt‐5’‐tiRNA‐Tyr. Our findings indicate that overexpression of mt‐5'‐tiRNA‐Tyr significantly elevates the succinylation level of HARS2 (Figure 5A). However, no lactylation or methylation modifications were observed on HARS2. Although acetylation levels also increased, their expression remained very low compared to succinylation (Figure S5A–C). Therefore, we conducted further studies on the succinylation modification of HARS2. Subsequently, we performed a quantitative analysis of succinylation modification sites, identifying two specific succinylation sites on HARS2 that are regulated by mt‐5’‐tiRNA‐Tyr (Figure 5B–D). To further elucidate the functional implications of these succinylation sites, we constructed HARS2 plasmids incorporating the designated mutations (K91R and K444R) (Figure 5E). Subsequent western blotting following IP revealed a significant reduction in the succinylation of HARS2‐K91R compared to the wild‐type HARS2 (WT) (Figure 5F). Comparative sequence analysis of HARS2 across various species indicated that the K91 lysine residue is conserved (Figure 5G). The molecular docking models depicting succinylation at the K91 site of HARS2 are presented in Figure 5H. Furthermore, the degradation rate of HARS2‐K91R was significantly faster than that of HARS2‐WT when treated with CHX (Figure 5I). Collectively, these findings suggest that K91 serves as the primary succinylation site for HARS2.

**FIGURE 5:**
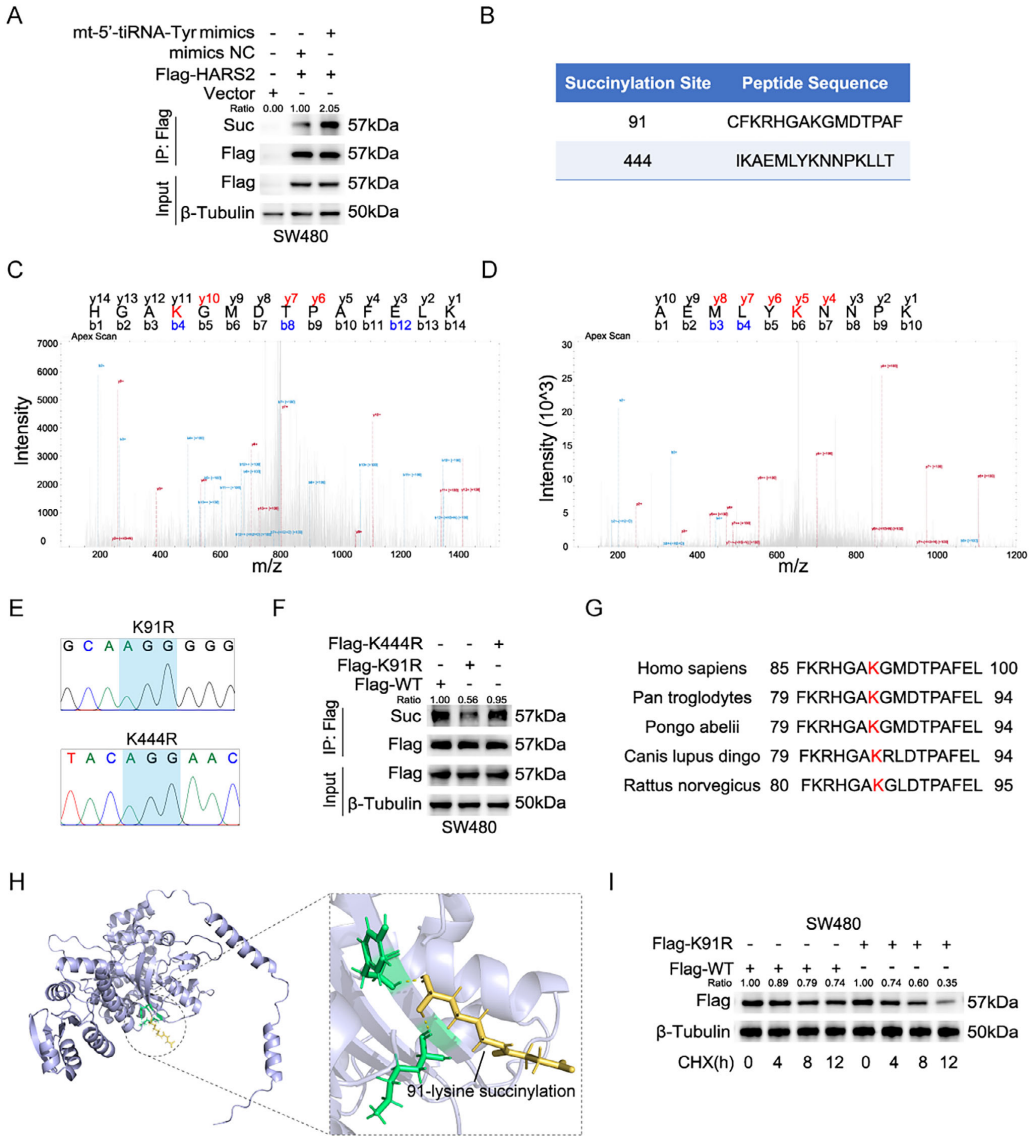
mt‐5’‐tiRNA‐Tyr increased the succinylation level of HARS2 at Lys91. (A) Co‐IP and western blotting were used to detect the succinylation level of HARS2. (B) The succinylation site and peptide sequence of HARS2 detected by succinylation and DIA proteomics. (C) MS/MS spectra of the succinylated HARS2 peptide for the identification and quantification of K91 succinylation on HARS2. (D) MS/MS spectra of the succinylated HARS2 peptide for the identification and quantification of K444 succinylation on HARS2. (E) Sanger sequencing was used to verify whether lysine (K) was mutated to arginine (R). (F) SW480 cells were transfected with the indicated plasmids, and the succinylation of HARS2 was detected by Co‐IP and western blotting. (G) Species conservation analysis of K91 sequence sites for HARS2. (H) The 3D structure of HARS2 K91 succinylation was predicted (Computed structure model of HARS2 predicted by AlphaFold). (I) SW480 cells were transfected with the indicated plasmids and then treated with CHX (100 µg/mL) for 0, 4, 8, and 12 h. The degradation rate of HARS2 was detected by western blotting.

### 2.8 Inhibition of Succinylation at Lysine 91 on HARS2 Results in Decreased Proliferation of CRC Cells

To examine the effects of desuccinylation at HARS2 K91 on CRC cell proliferation, we transfected HARS2‐WT or the HARS2 K91R mutant into stable sh‐HARS2 SW480 cells, which exhibit endogenous depletion of HARS2. The CCK‐8 and colony formation assays demonstrated a marked decrease in proliferation within the K91R group compared to the WT group (Figure 6A,B). Subsequently, the proliferation of HARS2 K91R cells was assessed using a CDX model. Xenografts derived from the K91R group showed a reduction in both tumor weight and volume when compared to the WT group (Figure 6C–E). No obvious difference in body weight was observed between the HARS2‐WT and HARS2‐K91R groups (Figure 6F). IHC further revealed a decrease in Ki67 and HARS2 expression, and an increase in Cleaved caspase‐3 expression, in the K91R group relative to the WT group (Figure 6G). These findings suggest that the inhibition of succinylation at lysine 91 in HARS2 leads to diminished proliferation of CRC cells.

**FIGURE 6:**
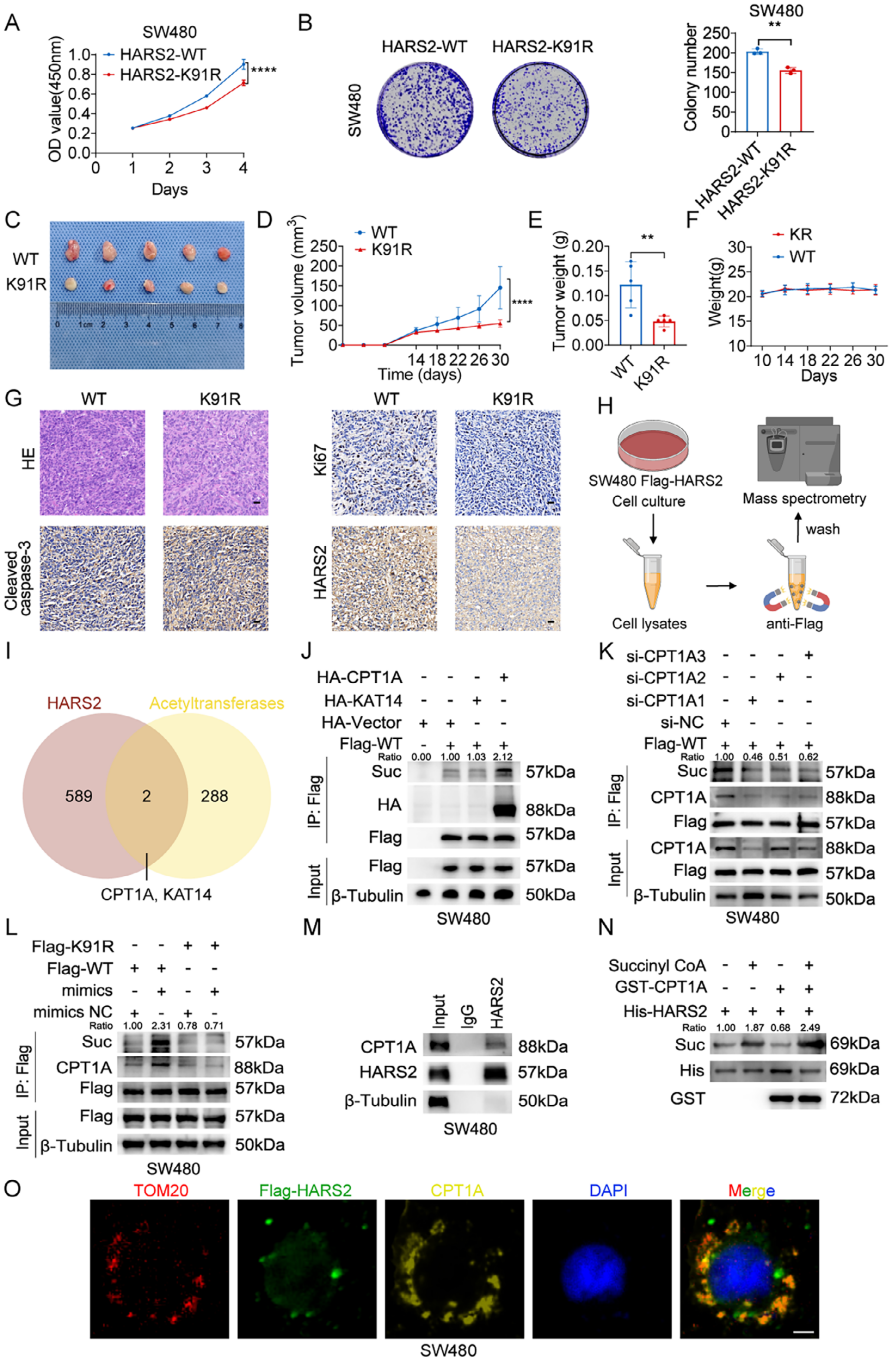
HARS2 K91 succinylation promotes CRC proliferation and is regulated by CPT1A. (A) CCK‐8 assays were performed to evaluate cell proliferation after transfection with the indicated plasmids (*n* = 3). ^****^ *p* < 0.0001. (B) Colony formation assays were performed to evaluate cell proliferation after transfection with the indicated plasmids (*n* = 3). ^**^ *p* < 0.01. (C–E) Tumor growth in the HARS2‐K91R group was suppressed, as shown by representative tumor images (C), tumor volume (D), and tumor weight (E), compared with the HARS2‐WT group (*n* = 5). ^****^ *p* < 0.0001, ^**^ *p* < 0.01. (F) Body weight of mice in the HARS2‐WT and HARS2‐K91R groups (*n* = 5). (G) HE staining and IHC analysis of Ki67, Cleaved caspase‐3, and HARS2 in xenograft tumors. Scale bar, 20 µm. (H, I) Schematic representation and Venn diagram of candidate HARS2‐interacting acyltransferases identified by IP‐MS. (J) CPT1A or KAT14 was overexpressed in SW480 cells, and HARS2 succinylation was detected by Co‐IP and western blotting. (K) HARS2 succinylation was detected after siRNA‐mediated knockdown of CPT1A by Co‐IP and western blotting. (L) The succinylation of HARS2 and the interaction between CPT1A and HARS2 were detected after transfection with mt‐5’‐tiRNA‐Tyr mimics by Co‐IP and western blotting. (M) Endogenous Co‐IP analysis of the interaction between CPT1A and HARS2 in SW480 cells. (N) In vitro reconstitution assay of HARS2 succinylation using purified recombinant His‐HARS2, GST‐CPT1A, and succinyl‐CoA. His‐HARS2 was added at 2.0 µg in each reaction, GST‐CPT1A was added at 0.25 µg where indicated, and succinyl‐CoA was added at 50 µm where indicated. HARS2 succinylation was detected by western blotting. (O) Immunofluorescence detection of the colocalization of TOM20, Flag‐HARS2, and CPT1A in SW480 cells. All values are means ± SD, and significance was determined by two‐way ANOVA followed by Tukey's test (A, D) or two‐tailed Student's *t*‐test (B, E).

To elucidate the regulators of HARS2 succinylation, we conducted IP followed by LC‐MS/MS in SW480 cells overexpressing HARS2 to identify potential HARS2 interactors (Figure 6H). The mass spectrometry analysis indicated that carnitine palmitoyltransferase 1A (CPT1A) and lysine acetyltransferase 14 (KAT14) may serve as candidate acyltransferases for HARS2 (Figure 6I). Co‐transfection experiments showed that CPT1A, but not KAT14, increased HARS2 succinylation (Figure 6J). Conversely, siRNA‐mediated knockdown of CPT1A reduced the succinylation level of HARS2 (Figure 6K). Western blotting analysis following IP further demonstrated that mt‐5’‐tiRNA‐Tyr mimics enhanced the interaction between CPT1A and HARS2‐WT, whereas this effect was diminished in HARS2‐K91R cells (Figure 6L). Endogenous Co‐IP confirmed the interaction between CPT1A and HARS2 in SW480 cells (Figure 6M). We further performed an in vitro reconstitution assay using purified recombinant His‐HARS2 and GST‐CPT1A proteins. Although succinyl‐CoA alone induced detectable HARS2 succinylation, the addition of recombinant GST‐CPT1A further increased the succinylation level of His‐HARS2 in the presence of succinyl‐CoA (Figure 6N). Immunofluorescence assays confirmed the mitochondrial colocalization of CPT1A and HARS2 (Figure 6O). These results suggest that mt‐5’‐tiRNA‐Tyr modulates HARS2 succinylation by influencing its interaction with CPT1A.

### 2.9 mt‐5’‐tiRNA‐Tyr Causes Mitochondrial Damage by Inhibiting Mitochondrial Translation

HARS2 serves as a crucial aminoacyl‐tRNA synthetase involved in mitochondrial protein translation. The absence of HARS2 has been associated with mitochondrial dysfunction [17]. However, the precise underlying mechanism remains unidentified. Western blotting analysis revealed that mt‐5’‐tiRNA‐Tyr mimics reduced the levels of mitochondria‐encoded proteins in SW480 and RKO cells (Figure 7A). To directly assess nascent mitochondrial translation, we performed HPG metabolic labeling in the presence of emetine to suppress cytosolic translation. The effectiveness of this assay condition was confirmed by detecting HPG‐labeled nascent protein signals under emetine treatment in both SW480 and RKO cells, with total protein staining used as a loading control (Figure S6). mt‐5’‐tiRNA‐Tyr mimics reduced HPG‐labeled nascent mitochondrial proteins in both CRC cell lines (Figure 7B). TEM analysis showed that mt‐5’‐tiRNA‐Tyr inhibition was associated with more preserved mitochondrial morphology and clearer cristae compared with the inhibitor NC group (Figure 7C). Previous studies have shown that Glu‐5'tsRNA‐CTC associates with LARS2 and reduces the interaction between LARS2 and mt‐tRNA‐Leu [18]. Therefore, we hypothesized that the inhibition of mitochondrial translation by mt‐5’‐tiRNA‐Tyr may be associated with altered HARS2‐RNA association and reduced HARS2‐mt‐tRNA‐His association. RIP assays showed that mt‐5’‐tiRNA‐Tyr mimics increased the association of HARS2 with mt‐5’‐tiRNA‐Tyr while decreasing the association of HARS2 with mt‐tRNA‐His (Figure 7D). In the HARS2‐K91R group, mitochondria‐encoded protein expression and HPG‐labeled nascent mitochondrial protein synthesis were increased compared with the HARS2‐WT group (Figure 7E,F). The HARS2‐K91R group also showed increased HARS2‐associated mt‐tRNA‐His and more intact mitochondrial morphology compared with the HARS2‐WT group (Figure 7G,H). Subsequent rescue experiments showed that HARS2 overexpression counteracted the inhibition of mitochondrial protein translation caused by mt‐5’‐tiRNA‐Tyr mimics (Figure 7I). Collectively, these findings suggest that mt‐5’‐tiRNA‐Tyr is associated with reduced HARS2‐mt‐tRNA‐His association and impaired nascent mitochondrial translation, and that this process is linked to HARS2 K91 succinylation.

**FIGURE 7:**
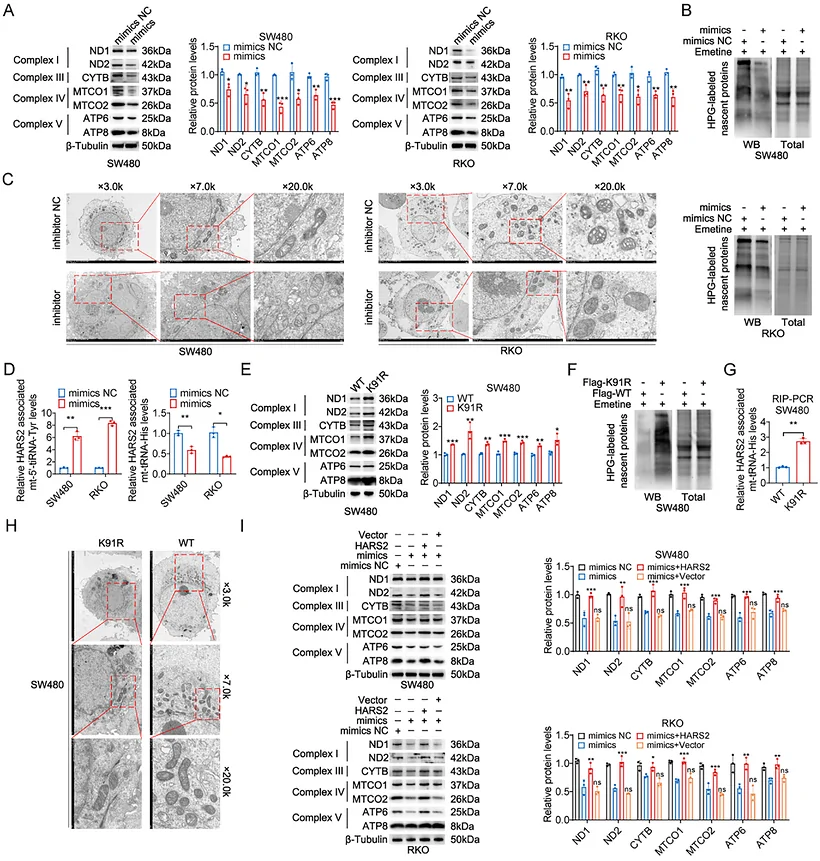
mt‐5’‐tiRNA‐Tyr causes mitochondrial damage by inhibiting mitochondrial translation. (A) Mitochondria‐encoded protein levels in SW480 and RKO cells after transfection with mt‐5’‐tiRNA‐Tyr mimics were detected by western blotting. ^***^ *p* < 0.001, ^**^ *p* < 0.01, ^*^ *p* < 0.05. (B) HPG‐based nascent mitochondrial protein synthesis assay in SW480 and RKO cells after transfection with mt‐5’‐tiRNA‐Tyr mimics in the presence of emetine. (C) Representative TEM images of SW480 and RKO cells after transfection with mt‐5’‐tiRNA‐Tyr inhibitor. (D) HARS2‐associated mt‐5’‐tiRNA‐Tyr and HARS2‐associated mt‐tRNA‐His levels were detected by RIP‐qPCR. ^***^ *p* < 0.001, ^**^ *p* < 0.01, ^*^ *p* < 0.05. (E) Mitochondria‐encoded protein levels in HARS2‐WT and HARS2‐K91R cells were detected by western blotting. ^***^ *p* < 0.001, ^**^ *p* < 0.01, ^*^ *p* < 0.05. (F) HPG‐based nascent mitochondrial protein synthesis assay in HARS2‐WT and HARS2‐K91R cells in the presence of emetine. (G) HARS2‐associated mt‐tRNA‐His levels in HARS2‐WT and HARS2‐K91R cells were detected by RIP‐qPCR. ^**^ *p* < 0.01. (H) Representative TEM images of HARS2‐WT and HARS2‐K91R cells. (I) Mitochondria‐encoded protein levels in mimics NC, mimics, mimics+HARS2, and mimics+Vector groups were detected by western blotting. ^***^ *p* < 0.001, ^**^ *p* < 0.01, ^*^ *p* < 0.05, nsP > 0.05. All values are means ± SD, and significance was determined by the two‐tailed Student's *t*‐test (A, D, E, G) or one‐way ANOVA followed by Tukey's test (I).

### 2.10 mt‐5’‐tiRNA‐Tyr Inhibits the Energy Metabolism of CRC Cells and Promotes the Formation of Succinate to Form a Positive Feedback Cycle

In tumor cells, mitochondrial damage can lead to impaired oxidative phosphorylation, resulting in a compensatory increase in aerobic glycolysis to sustain rapid tumor proliferation [19]. Consequently, we hypothesized that mitochondrial damage induced by mt‐5’‐tiRNA‐Tyr may influence tumor proliferation by modulating CRC metabolism. To investigate this, we conducted targeted energy metabolomics following the inhibition of mt‐5’‐tiRNA‐Tyr in SW480 cells (Figure 8A,B). The findings revealed reduced levels of α‐ketoglutaric acid, succinate, malate, lactate, and ATP after mt‐5’‐tiRNA‐Tyr inhibition (Figure 8C). We next examined OCR and glycolytic activity following transfection with the mt‐5’‐tiRNA‐Tyr inhibitor in SW480 cells. The inhibition of mt‐5’‐tiRNA‐Tyr increased basal and maximal respiration and reduced the glycolytic proton efflux rate (glycoPER), indicating that mt‐5’‐tiRNA‐Tyr promotes aerobic glycolysis while suppressing oxidative phosphorylation in CRC cells (Figure 8D). CCK‐8 and colony formation assays further showed that glycolysis inhibition by 2‐DG attenuated the proliferative effect induced by mt‐5’‐tiRNA‐Tyr mimics (Figure 8E,F). We then assessed succinate levels in CRC cells and observed a reduction after mt‐5’‐tiRNA‐Tyr inhibition, whereas mt‐5’‐tiRNA‐Tyr overexpression increased succinate levels (Figure 8G). Because succinate dehydrogenase (SDH) catalyzes succinate oxidation in the TCA cycle, we further measured SDH activity. SDH activity increased after mt‐5’‐tiRNA‐Tyr inhibition but decreased after mt‐5’‐tiRNA‐Tyr overexpression (Figure 8H), consistent with mt‐5’‐tiRNA‐Tyr‐dependent succinate accumulation. In Flag‐HARS2‐overexpressing SW480 cells, sodium succinate treatment increased HARS2 succinylation in a concentration‐dependent manner (Figure 8I). To further investigate whether succinate can promote CRC proliferation by enhancing HARS2 succinylation, colony formation and CCK‐8 assays were performed. Succinate supplementation rescued the inhibition of CRC cell proliferation caused by the mt‐5’‐tiRNA‐Tyr inhibitor (Figure 8J,K). A CDX model was then developed to evaluate the impact of succinate on CRC proliferation in vivo. Succinate supplementation rescued tumor growth, including tumor volume and tumor weight, in the mt‐5’‐tiRNA‐Tyr down group (Figure 8L–N). No obvious difference in body weight was observed among the NC, mt‐5’‐tiRNA‐Tyr down, and mt‐5’‐tiRNA‐Tyr down + succinate groups (Figure 8O). IHC analysis further showed increased Ki67 and HARS2 expression and decreased Cleaved caspase‐3 expression in the down + succinate group compared with the down group (Figure 8P). These findings suggest that mt‐5’‐tiRNA‐Tyr impairs mitochondrial protein translation, leading to mitochondrial dysfunction, which in turn shifts cellular energy metabolism from the tricarboxylic acid cycle to aerobic glycolysis. These findings support a model in which mt‐5’‐tiRNA‐Tyr impairs mitochondrial translation and reduces SDH activity, thereby contributing to succinate accumulation, which may further enhance HARS2 succinylation and promote CRC proliferation.

**FIGURE 8:**
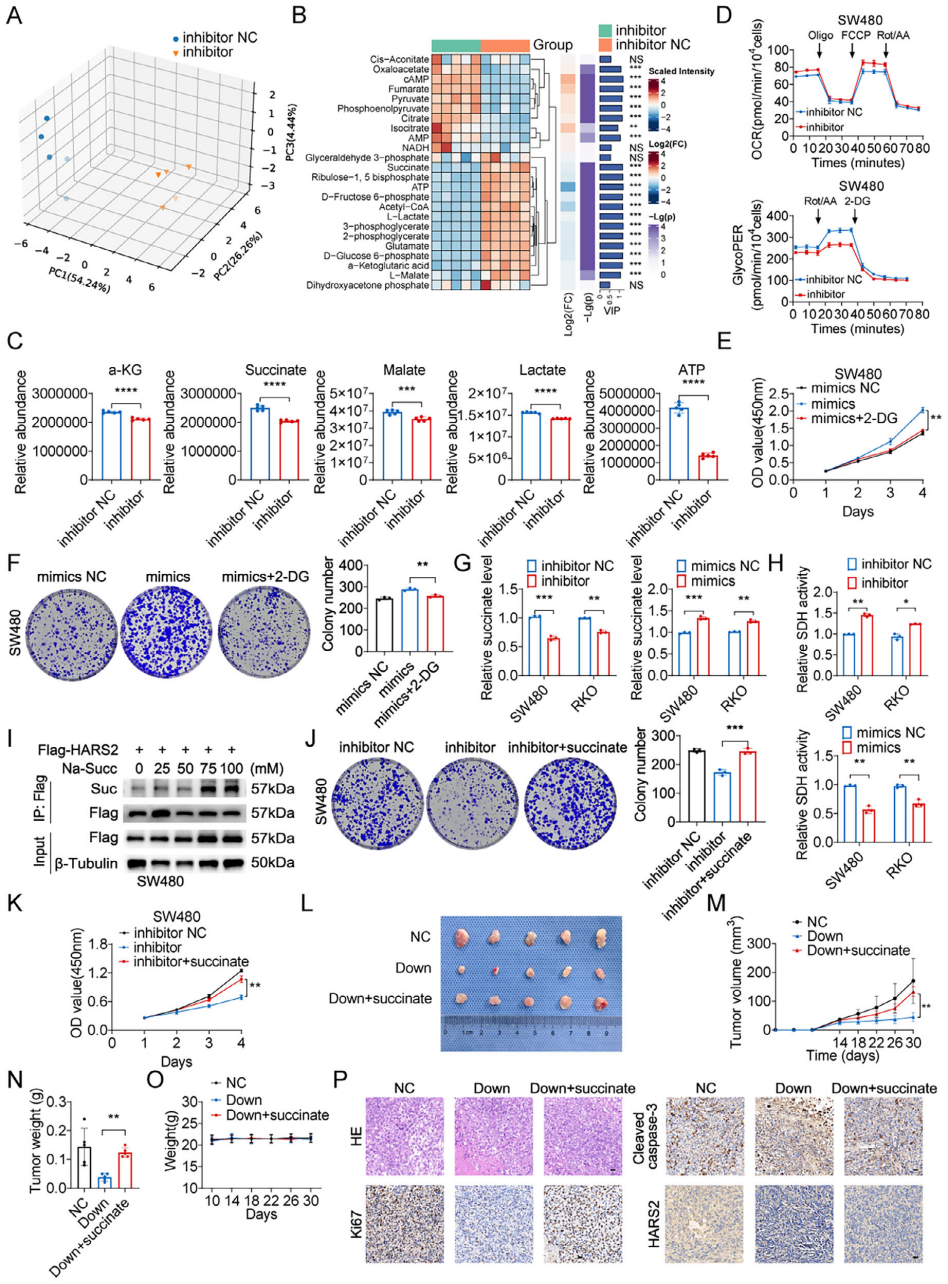
mt‐5’‐tiRNA‐Tyr inhibits energy metabolism in CRC cells and promotes succinate accumulation to form a positive feedback cycle. (A) PCA of metabolite expression in the inhibitor group compared with the inhibitor NC group (*n* = 5). (B) Heat map showing differentially abundant metabolites between the inhibitor and inhibitor NC groups (*n* = 5). (C) Relative abundance of core energy metabolites after mt‐5’‐tiRNA‐Tyr inhibition (*n* = 5). ^****^ *p* < 0.0001, ^***^ *p* < 0.001. (D) OCR and glycoPER in SW480 cells after transfection with mt‐5’‐tiRNA‐Tyr inhibitor (*n* = 3). (E, F) CCK‐8 and colony formation assays were performed to evaluate the effect of 2‐DG on mt‐5’‐tiRNA‐Tyr mimic‐induced proliferation (*n* = 3). ^**^ *p* < 0.01. (G) Relative succinate levels after transfection with mt‐5’‐tiRNA‐Tyr inhibitor or mimics. ^***^ *p* < 0.001, ^**^ *p* < 0.01. (H) SDH activity after transfection with mt‐5’‐tiRNA‐Tyr inhibitor or mimics. ^**^ *p* < 0.01, ^*^ *p* < 0.05. (I) HARS2 succinylation was detected after sodium succinate treatment by Co‐IP and western blotting. (J, K) Colony formation and CCK‐8 assays were performed to evaluate the effect of succinate supplementation on mt‐5’‐tiRNA‐Tyr inhibitor‐mediated proliferation (*n* = 3). ^***^ *p* < 0.001, ^**^ *p* < 0.01. (L–N) Tumor growth in the mt‐5’‐tiRNA‐Tyr down + succinate group was rescued, as shown by representative tumor images (L), tumor volume (M), and tumor weight (N), compared with the mt‐5’‐tiRNA‐Tyr down group (*n* = 5). ^**^ *p* < 0.01. (O) Body weight of mice in the NC, mt‐5’‐tiRNA‐Tyr down, and mt‐5’‐tiRNA‐Tyr down + succinate groups (*n* = 5). (P) HE staining and IHC analysis of Ki67, Cleaved caspase‐3, and HARS2 in xenograft tumors. Scale bar, 20 µm. All values are means ± SD, and significance was determined by the two‐tailed Student's *t*‐test (C, G, H), two‐way ANOVA followed by Tukey's test (E, K, M), or one‐way ANOVA followed by Tukey's test (F, J, N).

### 2.11 mt‐5’‐tiRNA‐Tyr Serves as a Plasma Biomarker for CRC

tsRNAs can be detected through non‐invasive blood tests, which are characterized by high sensitivity and specificity. To investigate the potential of mt‐5’‐tiRNA‐Tyr as a plasma biomarker for CRC, we analyzed its expression levels in a cohort consisting of 104 CRC patients, 50 patients with intestinal polyps, and 98 healthy donors. qRT‐PCR analysis demonstrated that plasma mt‐5’‐tiRNA‐Tyr expression was significantly elevated in CRC patients compared with both patients with intestinal polyps and healthy donors (Figure S8A). Moreover, mt‐5’‐tiRNA‐Tyr expression was markedly higher in both advanced‐stage and early‐stage CRC patients than in healthy donors (Figure S8B,C). Plasma mt‐5’‐tiRNA‐Tyr expression was also higher in stage III‐IV CRC than in stage I‐II CRC and higher in T3/T4 tumors than in T1/T2 tumors (Figure S8D,E). Post‐surgical analysis revealed a reduction in plasma mt‐5’‐tiRNA‐Tyr expression among CRC patients (Figure S8F).

We investigated the correlation between mt‐5’‐tiRNA‐Tyr and the clinically utilized tumor markers CEA, CA19‐9, CA72‐4, CA50, and CA24‐2. The expression level of mt‐5’‐tiRNA‐Tyr did not show a significant correlation with these conventional tumor markers (Figure S7), suggesting that plasma mt‐5’‐tiRNA‐Tyr may provide diagnostic information distinct from currently used clinical markers. We next evaluated the diagnostic performance of plasma mt‐5’‐tiRNA‐Tyr using ROC analysis with a training cohort and an internal validation cohort. CRC patients and healthy donors were randomly divided into a training cohort consisting of 73 CRC patients and 68 healthy donors and an internal validation cohort consisting of 31 CRC patients and 30 healthy donors. The combined panel was constructed using mt‐5’‐tiRNA‐Tyr together with CEA, CA19‐9, CA72‐4, CA50, and CA24‐2. In the training cohort, mt‐5’‐tiRNA‐Tyr achieved an AUC of 0.912 (95% CI, 0.858‐0.966), with a sensitivity of 0.836 and a specificity of 0.956 at the cutoff value of 3.452. The combined panel achieved an AUC of 0.937 (95% CI, 0.896‐0.977), with a sensitivity of 0.849 and a specificity of 0.912 (Figure S8G and Table S4). In the internal validation cohort, mt‐5’‐tiRNA‐Tyr retained diagnostic performance, with an AUC of 0.889 (95% CI, 0.794–0.984), a sensitivity of 0.710, and a specificity of 0.933. The combined panel achieved an AUC of 0.941 (95% CI, 0.887–0.995), with a sensitivity of 0.839 and a specificity of 0.933 (Figure S8H and Table S5). DeLong test comparisons showed that mt‐5’‐tiRNA‐Tyr performed significantly better than most conventional tumor markers, whereas the combined panel did not significantly outperform mt‐5’‐tiRNA‐Tyr alone in either the training or internal validation cohort. These internally validated analyses support the potential of plasma mt‐5’‐tiRNA‐Tyr as a diagnostic biomarker for CRC, while further external validation will be required in independent cohorts. For early‐stage CRC, mt‐5’‐tiRNA‐Tyr achieved an AUC of 0.873 and the combined panel achieved an AUC of 0.903 (Figure S8I). In distinguishing CRC patients from patients with intestinal polyps, mt‐5’‐tiRNA‐Tyr achieved an AUC of 0.820 and the combined panel achieved an AUC of 0.855 (Figure S8J). In summary, these findings support further evaluation of plasma mt‐5’‐tiRNA‐Tyr as a candidate diagnostic biomarker for CRC, pending validation in independent external cohorts.

## 3 Discussion

Increasing evidence indicates that translational regulation represents the most extensive molecular mechanism by which tsRNAs influence tumor progression. tsRNAs can either promote or inhibit the expression of various RNA‐binding proteins, thereby modulating protein biosynthesis through diverse mechanisms, and thus play a significant biological role in tumor progression [20]. For instance, it has been documented that tRF‐3022b interacts with galectin‐1 and macrophage migration inhibitory factor (MIF) in CRC cells, facilitating CRC progression by modulating MIF in M2 macrophages and reducing its polarization [21]. In our study, we utilized an RNA pull‐down assay to capture the binding protein of mt‐5’‐tiRNA‐Tyr, followed by mass spectrometry analysis, which revealed that HARS2 was the most abundant protein identified.

tRNA acts as an adapter that decodes mRNA into proteins within the ribosome‐dependent translation machinery. At the same time, aminoacyl‐tRNA synthetases (AARSs) are key enzymes responsible for attaching the correct amino acid to its corresponding tRNA [22, 23]. This process is crucial for the accuracy of protein translation. The specificity of this process is primarily determined by the interaction between AARS and its corresponding tRNA [23, 24]. This interaction is mediated through recognition elements within the tRNA, which are key sequences or structural features that the AARS can identify and bind to. Studies have shown that AARSs have relatively low specificity in amino acid activation and tRNA aminoacylation, with the binding of tRNA to AARSs, followed by the correct aminoacylation of tRNA, being dependent on specific nucleotides [22, 23, 25]. HARS2 is a crucial aminoacyl‐tRNA synthetase responsible for attaching histidine amino acids to mt‐tRNA‐His to form aminoacyl tRNA, a process essential for mitochondrial protein translation [26]. We further evaluated the association between mt‐5’‐tiRNA‐Tyr and HARS2 using RNA pull‐down/western blotting and RIP assays. Our findings indicated a significant reduction in HARS2 protein expression following the inhibition of mt‐5’‐tiRNA‐Tyr. Conversely, the overexpression of HARS2 was able to counteract the inhibitory effects of the mt‐5’‐tiRNA‐Tyr inhibitor on the proliferation of CRC cells. These results suggest that mt‐5’‐tiRNA‐Tyr may facilitate CRC cell proliferation by upregulating HARS2 expression levels.

PTMs serve as a core mechanism regulating protein function and diversity by chemically modifying specific amino acid residues on proteins after synthesis, such as phosphorylation, acetylation, methylation, ubiquitination, and SUMOylation [27]. PTMs significantly influence protein stability, spatial conformation, subcellular localization, interactions, and catalytic activity [16]. Consequently, they participate in regulating diverse biological processes, including cellular signaling, metabolic homeostasis, stress responses, and fate determination [16]. Within the gene expression regulatory network mediated by tsRNAs, PTMs play a pivotal role in modulating the expression and function of RNA‐binding proteins (RBPs) through tsRNAs [28, 29]. Typically, tsRNAs form complexes with specific RBPs to mediate alterations in their PTM status. Thus, systematically elucidating the role of protein PTMs in the tsRNA‐regulated RBP system holds significant importance. Research indicates that in papillary thyroid carcinoma, a 5'‐tRNA‐derived fragment named tRF‐1:30‐Gly‐CCC‐3 is significantly downregulated. It has been reported to associate with pyruvate carboxylase, promoting its ubiquitination and thereby inhibiting its stability, which disrupts the replenishment of TCA cycle intermediates and suppresses tumor cell proliferation and invasion [30]. In bladder cancer, mtiRL, an m7G‐modified tRNA‐derived small RNA regulated by METTL1, has been reported to associate with annexin A2 (ANXA2) and promote ANXA2 phosphorylation and nuclear translocation, thereby driving bladder cancer progression [28]. Utilizing Co‐IP and western blotting, we observed that mt‐5’‐tiRNA‐Tyr specifically facilitates the succinylation of HARS2. Succinylation involves the covalent attachment of succinyl groups to lysine residues, typically occurring on key enzymes within the central metabolic pathway, and plays a pivotal role in cellular metabolism [31, 32]. Hu et al. reported that the Succinate‐CoA Ligase GDP‐Forming Subunit β (SUCLG2) is overexpressed in lung adenocarcinoma. The knockout of SUCLG2 results in increased succinylation levels of mitochondrial proteins, thereby advancing the progression of lung adenocarcinoma [33]. Wang et al. demonstrated that S100 Calcium Binding Protein A10 (S100A10) undergoes succinylation at the K47 site mediated by CPT1A, which subsequently inhibits the ubiquitination of S100A10, thereby stabilizing its structure [34]. In our study, we utilized succinylation modification mass spectrometry to evaluate the expression levels of succinylated proteins following the overexpression of mt‐5’‐tiRNA‐Tyr. We then constructed HARS2 plasmids with Flag tags, incorporating specific mutations (K91R, K444R). Our results indicated that, compared to the HARS2‐WT, only the succinylation level of the HARS2‐K91R was reduced. Moreover, the stability of HARS2 was significantly compromised upon mutation at the K91 site. These findings suggest that mt‐5’‐tiRNA‐Tyr enhances the stability of the HARS2 protein by modulating succinylation at the K91 site. Although our data support the involvement of CPT1A in HARS2 succinylation, the precise mechanism by which mt‐5’‐tiRNA‐Tyr association with HARS2 is linked to this modification remains to be fully elucidated. One possible explanation is that mt‐5’‐tiRNA‐Tyr association with HARS2 may alter the local conformation of HARS2 or stabilize a HARS2 state that facilitates CPT1A association and K91 succinylation. However, the current data do not distinguish whether mt‐5’‐tiRNA‐Tyr alters K91 accessibility, promotes CPT1A recruitment, or acts through another structural mechanism. In addition, the attenuated CPT1A‐HARS2 interaction observed in HARS2‐K91R cells should not be interpreted as definitive evidence that K91 alone determines CPT1A substrate recognition. Further structural and biochemical studies will be required to clarify how mt‐5’‐tiRNA‐Tyr association with HARS2 is linked to CPT1A‐mediated HARS2 succinylation. A spatial issue that should be considered is that CPT1A is classically localized to the mitochondrial outer membrane, whereas mature HARS2 mainly functions in the mitochondrial matrix. Previous studies have demonstrated that CPT1A has lysine succinyltransferase activity and can regulate succinylation of multiple substrates, including cytosolic and mitochondrial proteins [35]. Therefore, CPT1A‐dependent succinylation is not necessarily restricted to proteins annotated within a single identical steady‐state submitochondrial compartment. Nevertheless, the precise mechanism by which CPT1A gains access to HARS2 in cells remains unresolved. It is possible that CPT1A acts on a subset of HARS2 during mitochondrial import or at mitochondrial membrane‐associated import/contact sites, or that additional adaptor proteins facilitate transient CPT1A‐HARS2 proximity. Although our data support the involvement of CPT1A in HARS2 succinylation, the spatial and enzymatic basis of this regulation remains incompletely resolved. The present in vitro reconstitution assay used wild‐type recombinant CPT1A, but did not include CPT1A catalytic mutants, mt‐5’‐tiRNA‐Tyr depletion/add‐back conditions, or mutant mt‐5’‐tiRNA‐Tyr controls. Future studies combining catalytic‐mutant reconstitution, RNA‐dependence assays, submitochondrial fractionation, protease‐protection assays, mitochondrial import assays, proximity labeling, and immuno‐electron microscopy will be required to determine the catalytic specificity, RNA dependence, and spatial mechanism of CPT1A‐associated HARS2 succinylation.

Mitochondria are essential organelles within the cell, playing a critical role in energy metabolism [36]. Research has demonstrated that in the brain, Glu‐5'tsRNA‐CTC interferes with the interaction between mt‐tRNA‐Leu and leucyl‐tRNA synthetase 2, thereby impairing the translation of mitochondria‐encoded proteins. This defective mitochondrial translation disrupts cristae organization, resulting in impaired glutaminase‐dependent glutamate production and diminished synaptosomal glutamate levels [18]. Nonetheless, the potential impact of tsRNAs on mitochondrial function in CRC remains unexplored. The results above suggested that mt‐5’‐tiRNA‐Tyr enhances the stability of HARS2, a pivotal aminoacyl‐tRNA synthetase involved in mitochondrial protein translation, through succinylation, and that increased stability may promote mitochondrial protein translation. Contrary to our initial expectations, the western blotting analysis revealed a decrease, rather than an increase, in the expression levels of mitochondria‐encoded proteins following the overexpression of mt‐5’‐tiRNA‐Tyr. This unexpected outcome prompted further investigation. Previous research indicates that Glu‐5'tsRNA‐CTC associates with LARS2 and reduces LARS2‐mt‐tRNA‐Leu association [18]. Therefore, we hypothesize that mt‐5’‐tiRNA‐Tyr may be associated with impaired mitochondrial protein translation through altered HARS2‐RNA association and reduced HARS2‐mt‐tRNA‐His association. Additionally, Xu et al. have previously demonstrated that the enhancement of Beclin1 K117me2 disrupts its interaction with BCL‐2 [37]. Drawing a parallel, we hypothesized that enhanced HARS2 K91suc might further reduce HARS2‐mt‐tRNA‐His association. Consistent with this hypothesis, our results showed that mt‐5’‐tiRNA‐Tyr overexpression decreased HARS2‐associated mt‐tRNA‐His. In addition, HARS2‐associated mt‐tRNA‐His increased after mutation of the succinylation site. Therefore, although HARS2 stability was enhanced, reduced HARS2‐mt‐tRNA‐His association was accompanied by impaired mitochondrial protein translation and mitochondrial dysfunction.

In tumors, mitochondrial damage leads to the inhibition of oxidative phosphorylation, compelling cells to enhance aerobic glycolysis as a compensatory mechanism to sustain rapid tumor growth [19]. Aerobic glycolysis plays a crucial role in tumor cell metastasis, not only supplying the energy necessary for rapid proliferation but also modifying the microenvironment to favor metastasis through the accumulation of lactate [38]. Our study demonstrated that the inhibition of mt‐5’‐tiRNA‐Tyr augmented the TCA cycle and reduced lactate production in CRC cells, as evidenced by targeted energy metabolism mass spectrometry. Concurrently, the level of succinate, an intermediate metabolite of the TCA cycle, was significantly diminished. This suggests that mitochondrial damage mediated by mt‐5’‐tiRNA‐Tyr induces a metabolic shift in CRC cells from the TCA cycle to aerobic glycolysis, further promoting the succinylation of HARS2 by enhancing succinate availability. This forms a positive feedback loop that facilitates the proliferation of CRC. We also acknowledge that the proposed positive feedback loop requires cautious interpretation. Although mt‐5’‐tiRNA‐Tyr inhibition decreased succinate levels and mt‐5’‐tiRNA‐Tyr overexpression increased succinate levels, metabolite abundance alone cannot fully define TCA cycle flux. By directly measuring SDH activity, we found that mt‐5’‐tiRNA‐Tyr inhibition increased SDH activity, whereas mt‐5’‐tiRNA‐Tyr overexpression decreased SDH activity, suggesting that succinate accumulation may result from impaired succinate oxidation rather than increased overall TCA cycle flux. However, further studies using isotope tracing and absolute metabolite quantification will be needed to determine whether endogenous succinate reaches a sufficient level to drive HARS2 succinylation under physiological conditions. Therefore, the succinate‐mediated positive feedback loop should be considered a supported mechanistic model rather than a fully resolved metabolic flux mechanism.

In clinical observations, plasma mt‐5’‐tiRNA‐Tyr expression was markedly elevated in CRC patients compared with healthy donors and patients with intestinal polyps, and its level decreased within seven days after CRC surgery. Using a training cohort and an internal validation cohort, we further evaluated its diagnostic performance. In the training cohort, mt‐5’‐tiRNA‐Tyr achieved an AUC of 0.912, and the combined panel incorporating mt‐5’‐tiRNA‐Tyr with established tumor markers achieved an AUC of 0.937. In the internal validation cohort, mt‐5’‐tiRNA‐Tyr retained good diagnostic performance, with an AUC of 0.889, while the combined panel achieved an AUC of 0.941. Notably, mt‐5’‐tiRNA‐Tyr also showed diagnostic potential for early‐stage CRC, with an AUC of 0.873, and distinguished CRC patients from patients with intestinal polyps with an AUC of 0.820. These findings support the potential of plasma mt‐5’‐tiRNA‐Tyr as a candidate diagnostic biomarker for CRC, while further validation in independent external cohorts will be required.

Several limitations of the discovery phase should be acknowledged. The initial PANDORA‐seq analysis was performed using tissues and plasma from only three CRC patients, which limited the statistical power for identifying differentially expressed tsRNAs. In addition, healthy donor plasma samples were not included in the plasma sequencing stage; therefore, the sequencing analysis could not directly identify CRC plasma enriched tsRNAs through a case‐control plasma discovery design. Instead, our candidate selection strategy was based on the intersection of tsRNAs upregulated in CRC tissues and highly abundant tsRNAs detected in CRC plasma, followed by qRT‐PCR validation in larger independent tissue and plasma cohorts. Therefore, the sequencing data should be interpreted as an exploratory screening step, whereas the clinical relevance of mt‐5’‐tiRNA‐Tyr is supported primarily by subsequent validation in larger cohorts. In addition, although we performed an internal validation analysis for the plasma biomarker cohort, an independent external validation cohort was not included in the present study. Future studies will include independent external cohorts, preferably from multiple centers, to further evaluate the diagnostic robustness and clinical applicability of plasma mt‐5’‐tiRNA‐Tyr. Another limitation of this study is that the proposed relationship between mt‐5’‐tiRNA‐Tyr, HARS2, and mt‐tRNA‐His was mainly supported by cellular RIP‐qPCR assays. Although these experiments showed that mt‐5’‐tiRNA‐Tyr was enriched in HARS2 immunoprecipitates and was associated with reduced HARS2‐bound mt‐tRNA‐His, we did not directly quantify the relative endogenous mitochondrial abundance of mt‐5’‐tiRNA‐Tyr and mt‐tRNA‐His. In addition, we did not perform purified biochemical reconstitution assays to determine binding affinity, stoichiometry, or displacement among recombinant HARS2, mt‐tRNA‐His, and mt‐5’‐tiRNA‐Tyr. Therefore, whether mt‐5’‐tiRNA‐Tyr alters HARS2‐mt‐tRNA‐His association under endogenous mitochondrial conditions requires further investigation using quantitative biochemical approaches. In addition, we did not directly measure HARS2 aminoacylation activity or mt‐tRNA‐His charging in this study. Although the [^3^H]‐histidine based mt‐tRNA‐His charging assay would provide direct evidence, this experiment requires a certified radioactive isotope handling facility that is currently not available in our laboratory. Therefore, our conclusion is limited to altered HARS2‐RNA association and impaired nascent mitochondrial translation, and future aminoacylation, tRNA charging, substrate‐binding, and enzyme kinetic assays will be required to determine whether K91 succinylation alters HARS2 catalytic activity. Thus, the present data support altered HARS2‐RNA association and impaired nascent mitochondrial translation at the cellular level, but they do not define changes in HARS2 catalytic aminoacylation activity.

In conclusion, mt‐5’‐tiRNA‐Tyr may contribute to impaired mitochondrial protein translation and mitochondrial damage through altered HARS2‐RNA association, reduced HARS2‐mt‐tRNA‐His association, and increased HARS2 succinylation, thereby prompting a metabolic shift in CRC from the TCA cycle to aerobic glycolysis. This shift results in the accumulation of succinate and establishes a positive feedback loop that enhances the malignant progression of CRC. This study evaluated the potential clinical relevance of plasma mt‐5’‐tiRNA‐Tyr in CRC and investigated its role in metabolic reprogramming through HARS2 succinylation. The findings are expected to provide both theoretical and experimental foundations for identifying novel biomarkers and potential therapeutic targets for CRC.

## 4 Experimental Section

### 4.1 PANDORA‐seq

Total RNA was isolated using the TRIZOL reagent (Invitrogen, Germany). Small RNAs ranging from 15 to 50 nucleotides were subjected to enzymatic processing to address junctional linkage issues arising from terminal modifications. Specific RNA methylation modifications were removed to facilitate the passage of reverse transcriptase. The sequencing data were analyzed using the Small RNA Annotation Software, SPORTS 1.1. After annotation by SPORTS1.1, the difference of tRNA fragments was analyzed by the R package DESeq2, and the screening conditions for significant difference tRNA fragments were as follows: Fold change > 2 and *p* < 0.05. For read length distribution analysis, reads mapped to the mt‐tRNA‐Tyr locus were extracted from the PANDORA‐seq annotation results, and the length of each mapped read was calculated. The proportion of the exact 31 nt mt‐5’‐tiRNA‐Tyr fragment was calculated as the number of reads corresponding to GGTAAAATGGCTGAGTGAAGCATTGGACTGT divided by the total number of reads mapped to the mt‐tRNA‐Tyr locus. The abundance of this exact fragment was further calculated as reads per million total tsRNA reads.

### 4.2 Human Specimens

A total of 48 pairs of CRC and adjacent tissues, along with microarrays from human CRC and adjacent tissues, were obtained from Nanjing First Hospital. All tissues were pathologically confirmed as CRC. Additionally, plasma samples were collected from 104 CRC patients, 98 healthy donors, 50 patients with intestinal polyps, and 32 postoperative CRC patients, between January 2022 and December 2023, at Nanjing First Hospital. All CRC patients were diagnosed and had not received any preoperative treatment. The study protocol received approval from the institutional ethics committee of Nanjing First Hospital, and informed consent was obtained from all participants (KY20220124‐04).

### 4.3 RNA Extraction, Reverse Transcription, and Quantitative Real‐Time PCR (qRT‐PCR)

Plasma total RNA was isolated utilizing the Smart 32 Nucleic Acid Extractor (DaAn Gene, China). In contrast, total RNA was extracted from tissues and cells using FreeZol Reagent (Vazyme Biotech Co., Ltd., China). Reverse transcription of tsRNA was performed using the stem‐loop method, incorporating specific reverse transcription primers. A total of 10 µL of complementary DNA (cDNA) was synthesized using the miRNA First Strand cDNA Synthesis Kit (Vazyme). For mt‐5’‐tiRNA‐Tyr detection, the stem‐loop reverse‐transcription primer contained a universal stem‐loop sequence and a short sequence complementary to the 3’ end of mt‐5’‐tiRNA‐Tyr, and qRT‐PCR was performed using an mt‐5’‐tiRNA‐Tyr‐specific forward primer and a universal reverse primer. The expected qRT‐PCR amplicon generated by the mt‐5’‐tiRNA‐Tyr‐specific forward primer and the universal reverse primer was 72 bp. For sequence validation, the qRT‐PCR product was cloned and then subjected to Sanger sequencing. qRT‐PCR was conducted on an ABI QuantStudio 5 system, employing a 20 µL reaction mixture comprising 10 µL of ChamQ Blue Universal SYBR qPCR Master Mix (Vazyme), 0.5 µL of primers (10 µm), five µL of enzyme‐free water, and four µL of cDNA. β‐actin and U6 served as internal reference genes for mRNA and tsRNA, respectively. Gene expression levels were quantified using the 2^−∆∆CT^ method. The sequences of all primers utilized are detailed in Table S1.

### 4.4 Northern Blot

Northern blot analysis was used to validate the endogenous size of mt‐5’‐tiRNA‐Tyr. Total RNA from CRC cell lines and paired tissue samples was separated on a denaturing polyacrylamide gel and transferred to a positively charged nylon membrane. The membrane was hybridized with a mt‐5’‐tiRNA‐Tyr‐specific probe according to the manufacturer's protocol (Beyotime, China). The signal was detected using a chemiluminescent detection system, and PAGE staining was used to evaluate RNA loading.

### 4.5 Mitochondrial and Cytosolic Fractionation

Mitochondrial and cytosolic fractions were prepared using a mitochondrial isolation kit (Beyotime) according to the manufacturer's protocol. mt‐5’‐tiRNA‐Tyr levels were quantified using the stem‐loop qRT‐PCR assay described above. MTATP8 and GAPDH were used as mitochondrial and cytosolic controls, respectively.

### 4.6 Cell Culture

The human CRC cell lines (SW480, RRID: CVCL_0546; RKO, RRID: CVCL_0504; HCT‐8, RRID: CVCL_2478; DLD‐1, RRID: CVCL_0248; HCT‐15, RRID: CVCL_0292; HCT‐116, RRID: CVCL_0291) and the human normal intestinal epithelial cell (NCM‐460, RRID: CVCL_0460) were procured from the ATCC (USA) or Chinese Academy of Science Cell Bank (Shanghai, China). All cell lines used in this study were acquired in 2020, authenticated through DNA sequencing using the STR method, and confirmed to be free of mycoplasma contamination. These cells were cultured in their respective medium (Procell, Wuhan, China), supplemented with 10% fetal bovine serum (Procell) and 1% penicillin‐streptomycin solution (HyClone, USA). The culture medium was replaced every two days, and the cells were maintained in an incubator at 37°C with 5% CO_2_.

### 4.7 Plasmids and Lentiviral Infection

HA‐CPT1A and HA‐KAT14 were obtained from the MiaoLing Plasmid Platform (Wuhan, China). Constructs for the WT, K91R, and K444R variants of HARS2, the promoter of Angiogenin (ANG), and siRNA targeting ANG were acquired from GeneAdv Co., Ltd. (Suzhou, China) and utilized according to the manufacturer's protocols. Additionally, mt‐5’‐tiRNA‐Tyr down, shRNAs targeting HARS2, HARS2 WT, HARS2 K91R, and empty vectors were acquired from GeneChem (Shanghai, China). The mt‐5’‐tiRNA‐Tyr down construct was designed as a lentiviral sponge/decoy construct containing tandem antisense binding units targeting mt‐5’‐tiRNA‐Tyr separated by short linker sequences. This construct was used to reduce the functional availability of mt‐5’‐tiRNA‐Tyr rather than as a conventional shRNA. Lentiviral particles were employed to infect SW480 cells, establishing stable cell lines using puromycin as a selection marker.

### 4.8 Cell Transfection

The synthesis of the inhibitor and mimics of mt‐5’‐tiRNA‐Tyr was conducted by Sangon Biotech (Shanghai, China). The sequence information is provided in Table S2. Cell transfections were performed using the Lipofectamine 3000 Transfection Reagent (Thermo Fisher Scientific). Cells were cultured to reach 70%–80% confluency before transfection. Initially, the Lipofectamine 3000 reagent was diluted in Opti‐MEM medium. Subsequently, the DNA was diluted in Opti‐MEM medium, followed by the addition of the P3000 Reagent. The diluted DNA was then combined with the diluted Lipofectamine 3000 Reagent in a 1:1 ratio and incubated for 15 min. Finally, the DNA‐lipid complexes were introduced to the cells. The P3000 Reagent was not required for the transfection of siRNA, inhibitors, or mimics.

### 4.9 CCK‐8

After a 36–48‐h transfection period, the cells were collected and plated into 96‐well plates at a density of 3 × 10^3^ cells per well. Once the cells adhered, the CCK‐8 reagent (Beyotime) was introduced, and the plates were incubated at 37°C for about 2 h. Absorbance was then measured at wavelengths of 450 nm using a microplate reader. These measurements were conducted every 24 h for a period of three days.

### 4.10 Colony Formation Assay

After transfection for 36–48 h, 1000 cells were plated into 6‐well plates. The culture medium was replaced every four days for approximately 12 days. At the conclusion of this incubation period, the cells were fixed at room temperature using 4% paraformaldehyde (Biosharp, China), stained with crystal violet, and subsequently photographed.

### 4.11 Animal Experiments

The Animal Management Committee of Nanjing First Hospital approved the animal experimental protocols utilized in this study (DWSY‐25131764). All animal procedures were conducted in strict compliance with ethical guidelines for animal experimentation. Eight‐week‐old female BALB/c nude mice with an initial body weight of 18–22 g were used for the xenograft experiments, and no obvious body weight fluctuation was observed during the experimental period. Body weight was recorded every three days during the xenograft experiment. Stable NC, mt‐5’‐tiRNA‐Tyr down, HARS2‐WT, and HARS2 K91R SW480 cells (1 × 10^7^ cells in 100 µL DMEM medium) were subcutaneously injected into the right axilla of BALB/c nude mice (5 mice per group). Tumor volume was calculated every three days using the formula: volume = (length × width^2^) / 2.

### 4.12 RNA Pull‐Down

An RNA pulldown assay for mt‐5’‐tiRNA‐Tyr‐binding proteins was modified using the Pierce Magnetic RNA‐Protein Pull‐Down Kit. Biotin‐labeled mt‐5’‐tiRNA‐Tyr sense and antisense were synthesized by GeneAdv. 50 µL of streptavidin Dynabeads were resuspended in 200 µL of binding buffer and then mixed with mt‐5’‐tiRNA‐Tyr sense and antisense RNA. Then, the mixtures were rotated for 0.5 h at 25°C. 2 mg of protein lysate from SW480 cells was added to the tubes, which were rotated for two h at 4°C. After washing three times on a magnetic separation rack, RNA‐protein complexes were resuspended in wash buffer and denatured with SDS‐PAGE loading buffer at 100°C for 10 min.

### 4.13 RNA Immunoprecipitation (RIP) Assay

SW480 cells underwent RIP using the Magna RIP RNA‐Binding Protein Immunoprecipitation Kit (Millipore, USA). The cells were lysed in RIP lysis buffer supplemented with a protease inhibitor cocktail and an RNase inhibitor. Subsequently, 50 µL of protein A/G magnetic beads were washed and combined with 5 µg of antibody. Following a gentle rotation for 2 h at 4°C, the antibody‐bound magnetic beads were isolated and incubated with the cell lysates overnight at 4°C. The antibody‐protein‐RNA complexes were then separated using a magnetic stand. RNA was extracted and quantified via qRT‐PCR as previously described.

### 4.14 Dual‐Luciferase Reporting Assay

Cells were seeded in 24‐well plates and transfected with either the relevant plasmids or NC. After 48 h, the cells were harvested and lysed with a lysis solution. The luciferase activity was then measured using the Dual–Luciferase Reporter Assay System (Promega, USA) according to the instructions.

### 4.15 In Situ Hybridization (ISH) and Fluorescence ISH (FISH)

ISH was executed using an hsa‐mt‐5’‐tiRNA‐Tyr probe provided by Servicebio (Wuhan, China). According to the protocol, the FISH assay was performed to detect the mt‐5’‐tiRNA‐Tyr probe signal in SW480 cells using the Ribo FISH Kit (RiboBio, China). The mt‐5’‐tiRNA‐Tyr was synthesized by Sangon Biotech.

### 4.16 Immunofluorescence

For immunofluorescence colocalization assays, cells were fixed with 4% paraformaldehyde, permeabilized, blocked, and incubated with the indicated primary antibodies overnight at 4°C. After washing, cells were incubated with fluorescent secondary antibodies and counterstained with DAPI. Images were captured using a fluorescence or confocal microscope. TOM20 was used as a mitochondrial marker for colocalization analysis.

### 4.17 Oxygen Consumption Rate (OCR) and Glycolysis Rate Assay

10 000 SW480 cells were cultured in a Seahorse 96‐well assay plate. After an overnight culture, the cells were washed twice with the prepared assay solution, and the pretreated probe plate was placed in the cell plate. OXPHOS or glycolysis rate assay (Agilent Technologies) reagents were added sequentially, and the cells were analyzed with the Agilent Seahorse XFe96 (Agilent Technologies).

### 4.18 Western Blotting

Cellular total proteins were extracted using RIPA lysis buffer supplied by SolarBio Life Sciences (Beijing, China), according to the manufacturer's instructions. The proteins were denatured by heating at 100°C for 10 min. Subsequently, an equivalent quantity of protein (30 µg) from each sample was separated by SDS‐PAGE and then transferred to polyvinylidene fluoride membranes (Millipore, USA). To prevent non‐specific binding, the membranes were incubated with QuickBlock Protein‐Free Blocking Buffer for Western Blot (Beyotime). Primary antibodies were applied and incubated overnight at 4°C. On the following day, horseradish peroxidase (HRP)‐conjugated secondary antibodies were added and incubated for 2 h at room temperature. Protein expression was detected using the enhanced chemiluminescence (ECL) method, with β‐Tubulin serving as an internal reference. All antibodies utilized in this study are detailed in Table S3. The relative ratio of the target protein was determined as the expression level of the target protein normalized to the internal control, with protein expression quantified using Image J software.

### 4.19 HPG‐Based Nascent Mitochondrial Protein Synthesis Assay

Nascent mitochondrial protein synthesis was evaluated using an HPG‐based nascent protein synthesis assay kit (Beyotime) according to the manufacturer's protocol. Briefly, cells were incubated in methionine‐free medium and treated with emetine to inhibit cytosolic translation before HPG labeling. The click reaction and detection were performed according to the kit instructions. HPG‐labeled nascent mitochondrial proteins were analyzed by western blotting, and total protein staining was used as a loading control.

### 4.20 Immunohistochemistry (IHC)

Immunohistochemistry (IHC) was performed on paraffin‐embedded sections of animal tumors using the IHC kit provided by Servicebio (Wuhan, China), according to the manufacturer's protocol. Primary antibodies specific to HARS2, Ki67, and Cleaved caspase‐3 were employed in the analysis.

### 4.21 Metabolomics Analysis

Metabolomics analysis was performed by Shanghai Bioprofile Technology Co., Ltd.

Metabolites were extracted from the cellular residue using 1 mL of a precooled methanol/acetonitrile/water solution (v/v, 2:2:1) under sonication for 1 h in ice baths. The resulting mixture was incubated at ‐20°C for 1 h, followed by centrifugation at 14 000 g and 4°C for 20 min. The supernatant was then transferred to sampling vials for subsequent LC‐MS analysis. To ensure the quality of the metabolic profiling data, quality control (QC) samples were prepared by pooling aliquots representative of all samples under analysis and were utilized for data normalization. These QC samples were prepared and analyzed using the same protocol as the experimental samples in each batch. The dried extracts were reconstituted in 50% acetonitrile, filtered through a disposable 0.22 µm cellulose acetate filter, transferred into 2 mL HPLC vials, and stored at ‐80°C until analysis.

### 4.22 Immunoprecipitation (IP)

Cells were lysed in an ice‐cold IP buffer supplemented with a protease inhibitor cocktail (Thermo Fisher Scientific) and a deacetylase inhibitor (MCE, Shanghai, China). The IP procedure was conducted by incubating Flag magnetic beads (NuoyiBio, Tianjin, China) with the lysate at 4°C for 2 h. Following incubation, the beads underwent three washes with ice‐cold IP buffer. Subsequently, proteins bound to the magnetic beads were eluted by heating at 95°C for 10 min in loading buffer (New Cell & Molecular Biotech, Suzhou, China).

### 4.23 In Vitro HARS2 Succinylation Assay

For the in vitro reconstitution assay, purified recombinant HARS2 protein (2 µg) was incubated with succinyl‐CoA (50 µm) in the presence or absence of purified recombinant CPT1A protein (0.25 µg) in a 50 µL reaction system containing HEPES reaction buffer, MgCl2, and DTT. The reaction was performed at 30°C for 2 h and terminated by adding SDS loading buffer. HARS2 succinylation was detected by western blotting.

### 4.24 Succinylation DIA Proteomics

Succinylation DIA proteomics were performed by Shanghai Bioprofile Technology Co., Ltd.

#### 4.24.1 Succinylation, Proteomics Sample Preparation

Succinyl‐modified peptides were enriched using the PTMScan Succinyl‐Lysine Motif Kit, while ubiquitin‐modified peptides were enriched using their respective kit. Reconstituted peptides were incubated with antibody‐linked Protein‐A agarose beads, washed with IPA buffer, and eluted with 0.15% TFA. The modified peptides were then desalted using a C18 Stage Tips column.

#### 4.24.2 LC‐MS Analysis

LC‐MS/MS analysis was conducted using an Orbitrap Astral mass spectrometer with a Vanquish Neo UHPLC system. Peptides were loaded onto a 50 cm Low‐Load µPAC Neo HPLC Column at 2.2 µL/min. The mobile phases were 0.1% formic acid in water (A) and 0.1% formic acid in 80% acetonitrile (B). Peptides were eluted over 8 min with a gradient of buffer B at 1.25 µL/min: 4% to 6% (0–0.1 min), 6%–12% (0.1–1.1 min), 12%–25% (1.1–4.3 min), 25%–45% (4.3–6.1 min), 45%–99% (6.1–6.5 min), and held at 99% (6.5–8 min). Peptides were analyzed using an Orbitrap Astral mass spectrometer. The DIA method included a 380–980 m/z survey scan at 240 000 resolution, with a 500% AGC target and an injection time of 5 ms. DIA MS/MS scans ranged from 150–2000 m/z with a 2 m/z isolation window, 500% AGC target, and 3 ms injection time. The normalized collision energy was 25, with a cycle time of 0.6s. Full MS and DIA scans were recorded in profile and centroid modes, respectively.

### 4.25 Succinate Measurement

After transfection, intracellular succinate levels were measured using the Amplex Red Succinate Assay Kit (Beyotime) according to the manufacturer's protocol.

### 4.26 SDH Activity Assay

Succinate dehydrogenase (SDH) activity was measured using an SDH activity assay kit (Beyotime) according to the manufacturer's protocol. Cells were collected after the indicated treatments, and the assay was performed using freshly prepared cell lysates. Absorbance was measured with a microplate reader, and SDH activity was normalized to the corresponding protein concentration.

### 4.27 Statistical Analysis

Statistical analysis was conducted utilizing IBM SPSS Statistics version 20.0 and GraphPad Prism version 9. For data exhibiting a normal distribution, results are expressed as mean values ± standard deviation (SD). Conversely, data not conforming to a normal distribution across groups are presented as medians accompanied by interquartile ranges. For normally distributed datasets, statistical evaluations were performed using the t‐test, one‐way ANOVA, or two‐way ANOVA. In contrast, datasets with non‐normal distributions were analyzed using the Mann‐Whitney U test, Kruskal–Wallis test, or Wilcoxon test. The area under the curve (AUC) was employed to assess the diagnostic efficacy of plasma mt‐5’‐tiRNA‐Tyr. For the biomarker analysis, CRC patients and healthy donors were randomly divided into a training cohort and an internal validation cohort. The combined diagnostic panel was constructed using a logistic regression model incorporating mt‐5’‐tiRNA‐Tyr, CEA, CA19‐9, CA72‐4, CA50, and CA24‐2. Cut‐off values were determined in the training cohort and applied to the internal validation cohort. The AUC, 95% CI, sensitivity, specificity, and accuracy were calculated, and DeLong tests were used to compare the AUCs between mt‐5’‐tiRNA‐Tyr and the conventional tumor markers or the combined panel. Spearman correlation analysis was used to evaluate the association between plasma mt‐5’‐tiRNA‐Tyr expression and conventional clinical tumor markers.

All experiments were repeated three times, and statistical significance was determined at a threshold of *p* < 0.05.

## Author Contributions

G.X. wrote the first draft of the manuscript. G.X. designed and supervised the entire project. G.X. performed most of the experiments. Z.D., L.X., H.S., and Y.X. conducted partial experiments. N.J., X.T., and P.Y. contributed to the clinical samples. X.M., S.H., and W.S. provided guidance and resources for the paper. All authors read and approved the final manuscript.

## Ethics Statement

The studies involving human participants were reviewed and approved by the Ethics Committees and Institutional Review Boards of Nanjing First Hospital, affiliated with Nanjing Medical University. The patients/participants provided their written informed consent to participate in this study. Written informed consent was obtained from the individuals for the publication of any potentially identifiable images or data included in this article (KY20220124‐04). The Animal Management Committee of Nanjing First Hospital approved the animal experimental protocols utilized in this study (DWSY‐25131764).

## Conflicts of Interest

The authors declare no conflicts of interest.

## Supporting information




**Supporting File 1**: advs77042‐sup‐0001‐SuppMat.docx.

**Supporting File 2**: advs77042‐sup‐0002‐DataSet.pdf.

## Data Availability

The data used in the current study are available from the corresponding author upon reasonable request.
