# Supplementary material for: A Novel tRNA Half mt‐5’‐tiRNA‐Tyr Promotes Colorectal Cancer Proliferation via Inducing HARS2 Succinylation
Source: Adv Sci (Weinh). 2026 Aug 21;13(57):e77042. doi: 10.1002/advs.77042 (PMC13496151; doi:10.1002/advs.77042)

Figure 2F

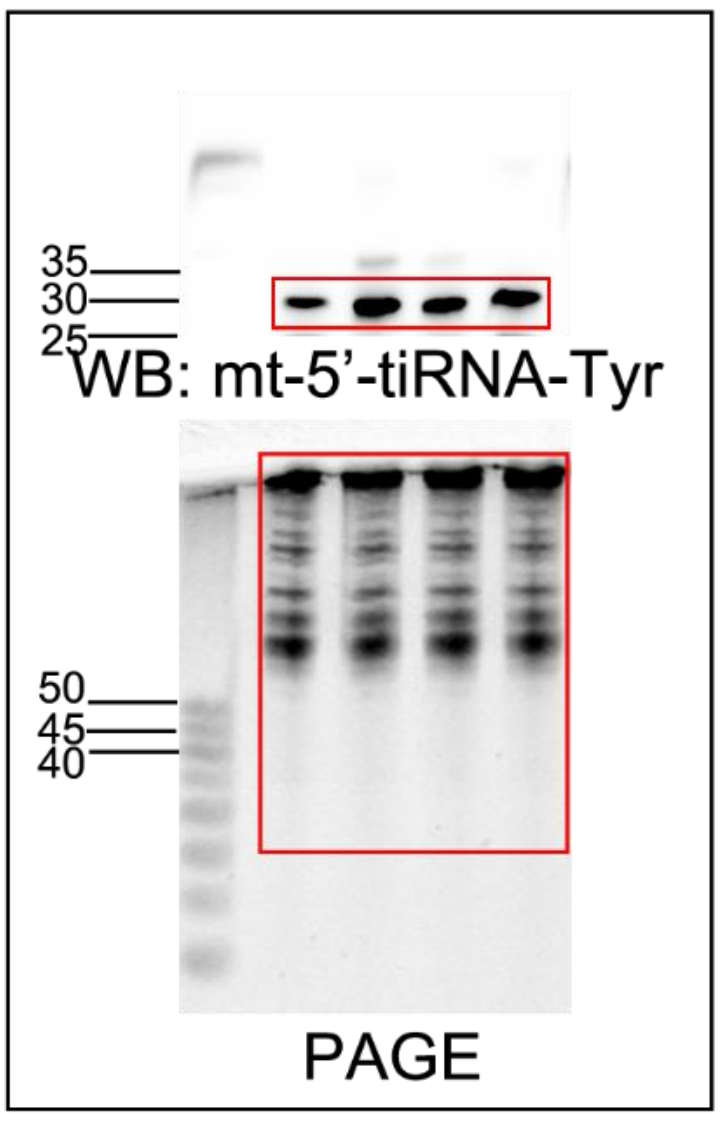

Figure 2G

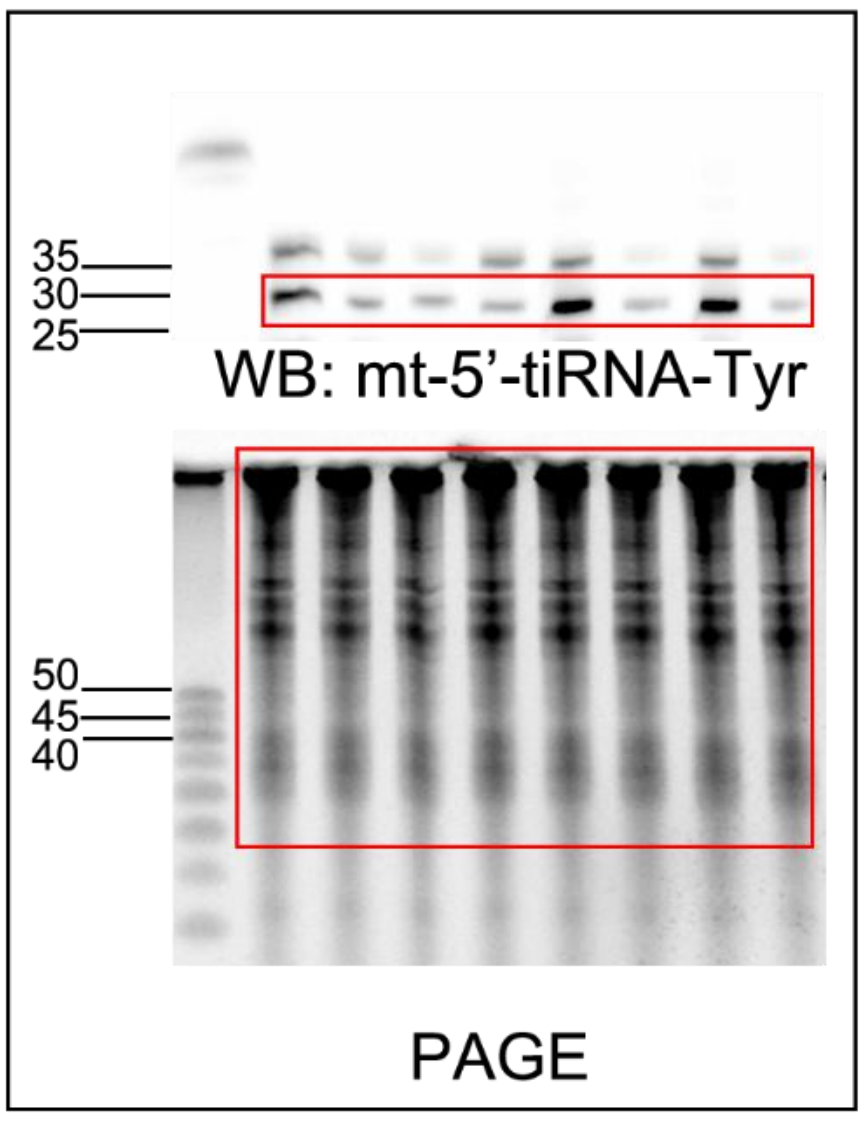

Figure 4F

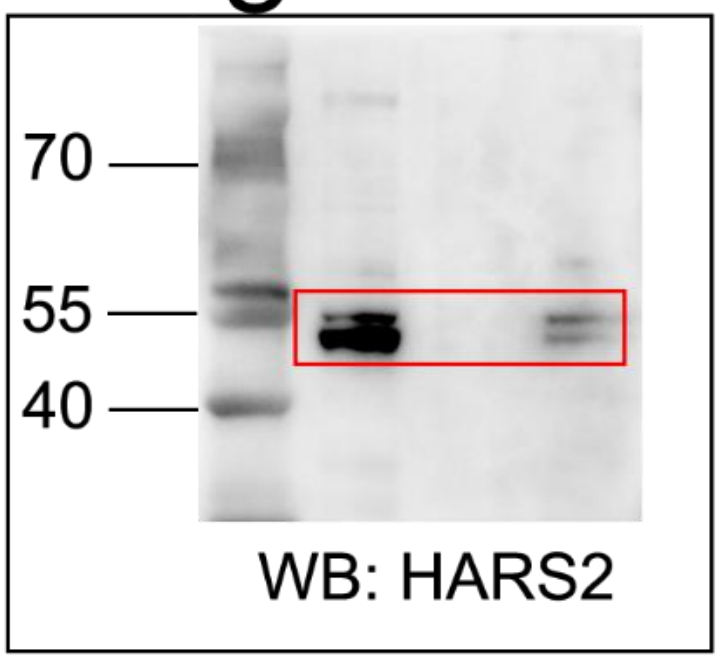

Figure 4I

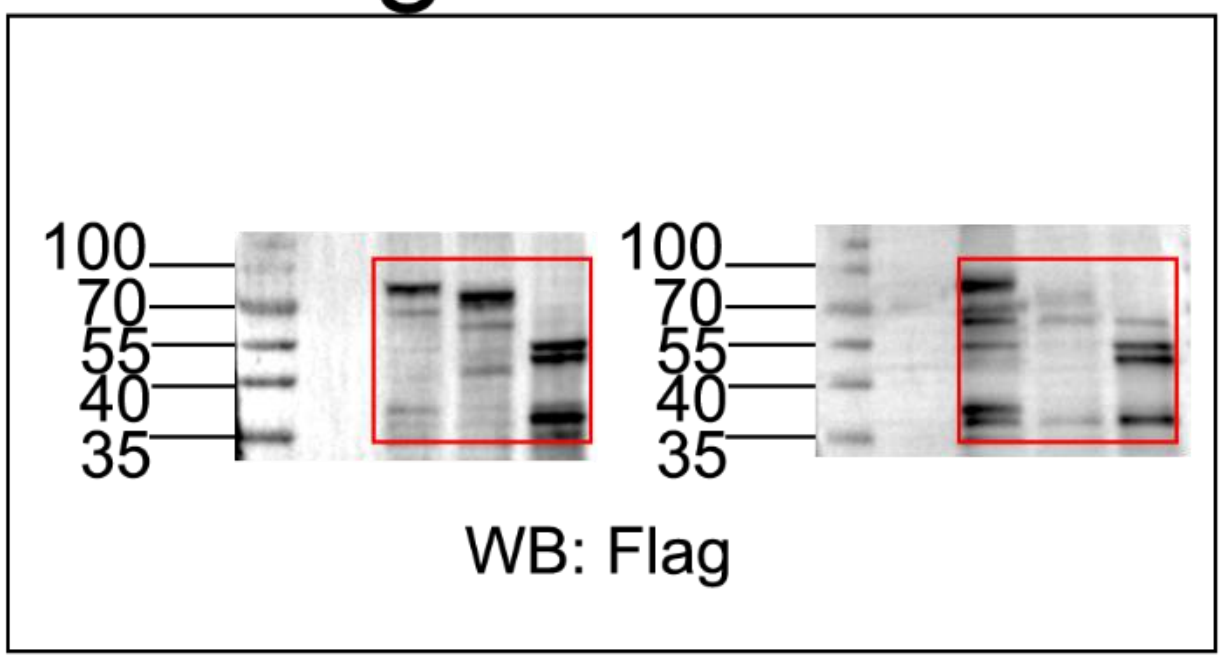

Figure 4M

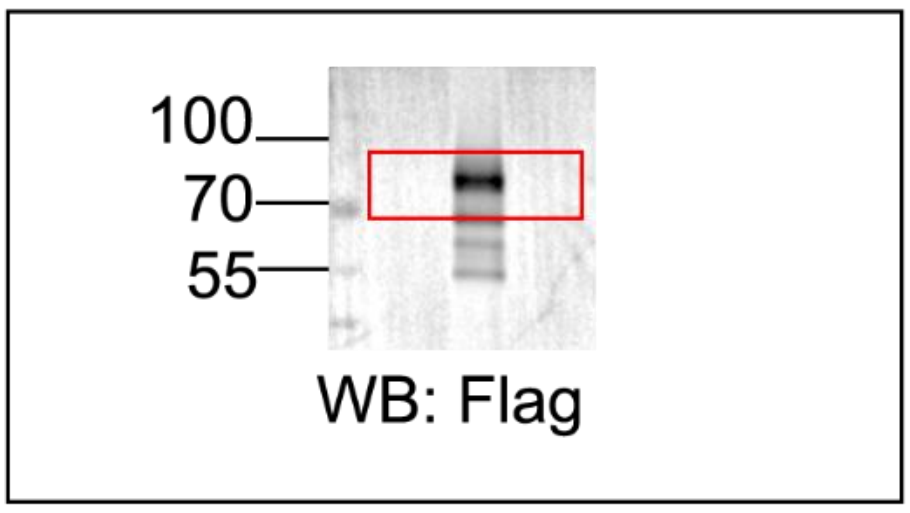

Figure 5A

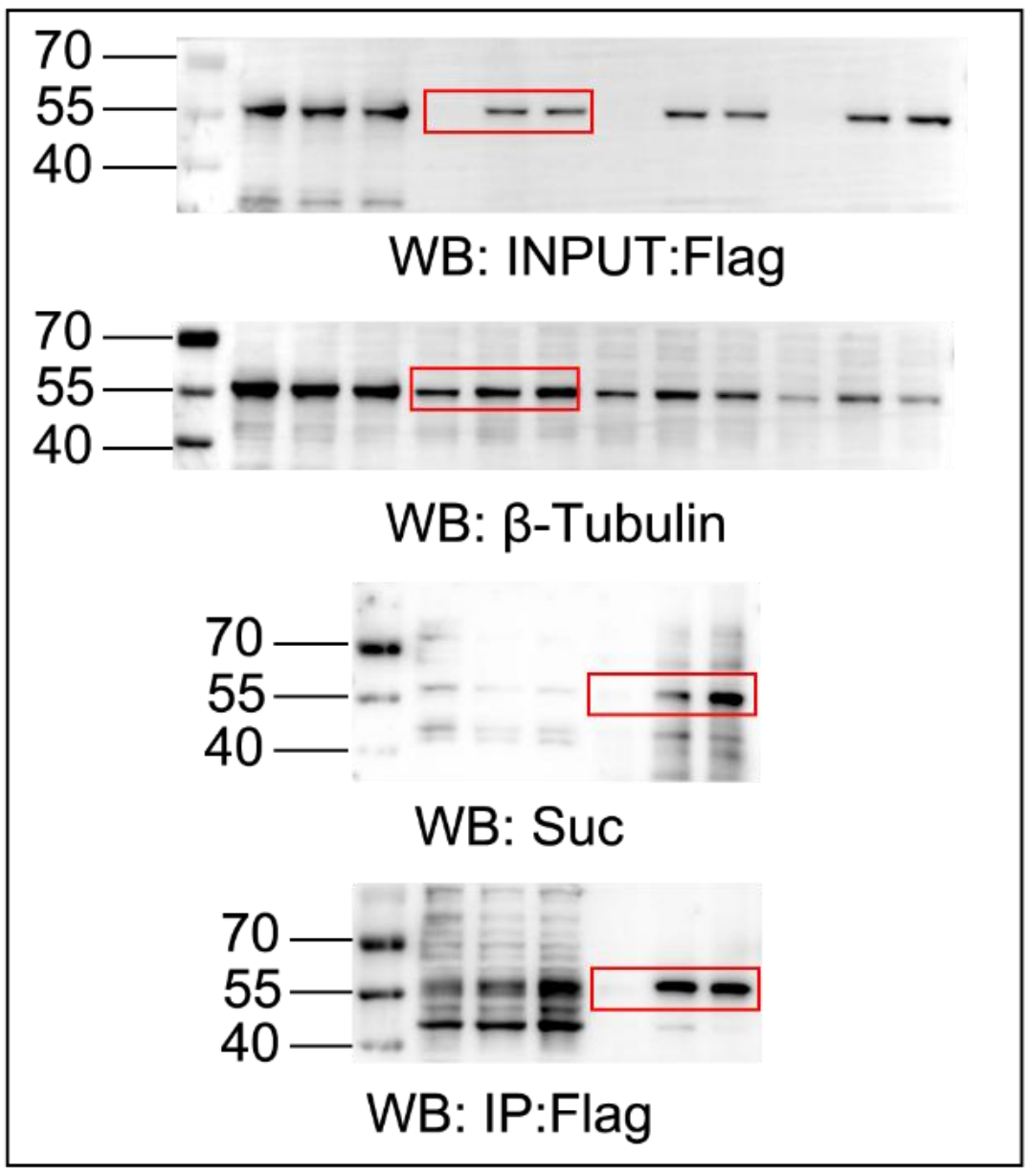

Figure 5F

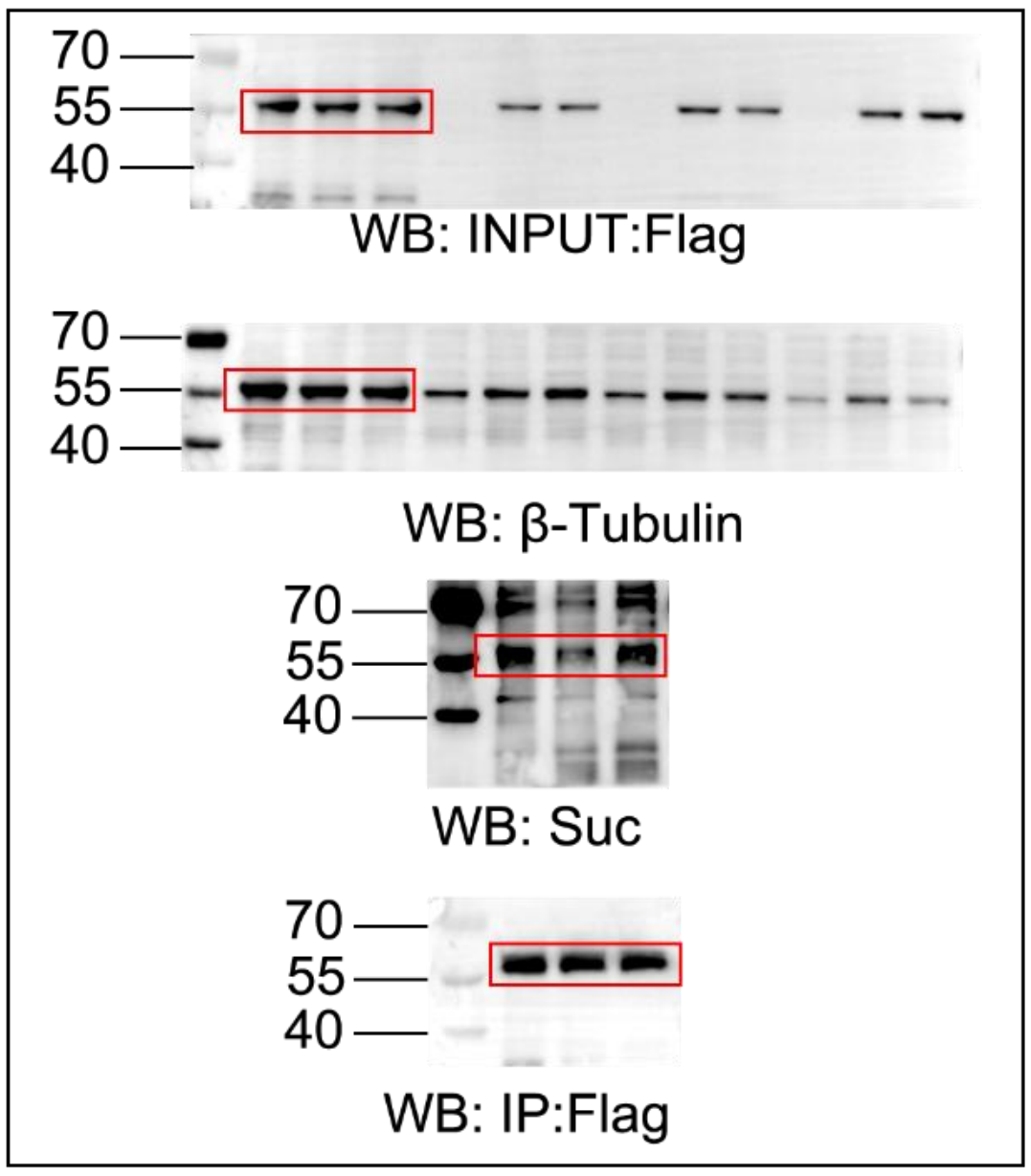

Figure 6J

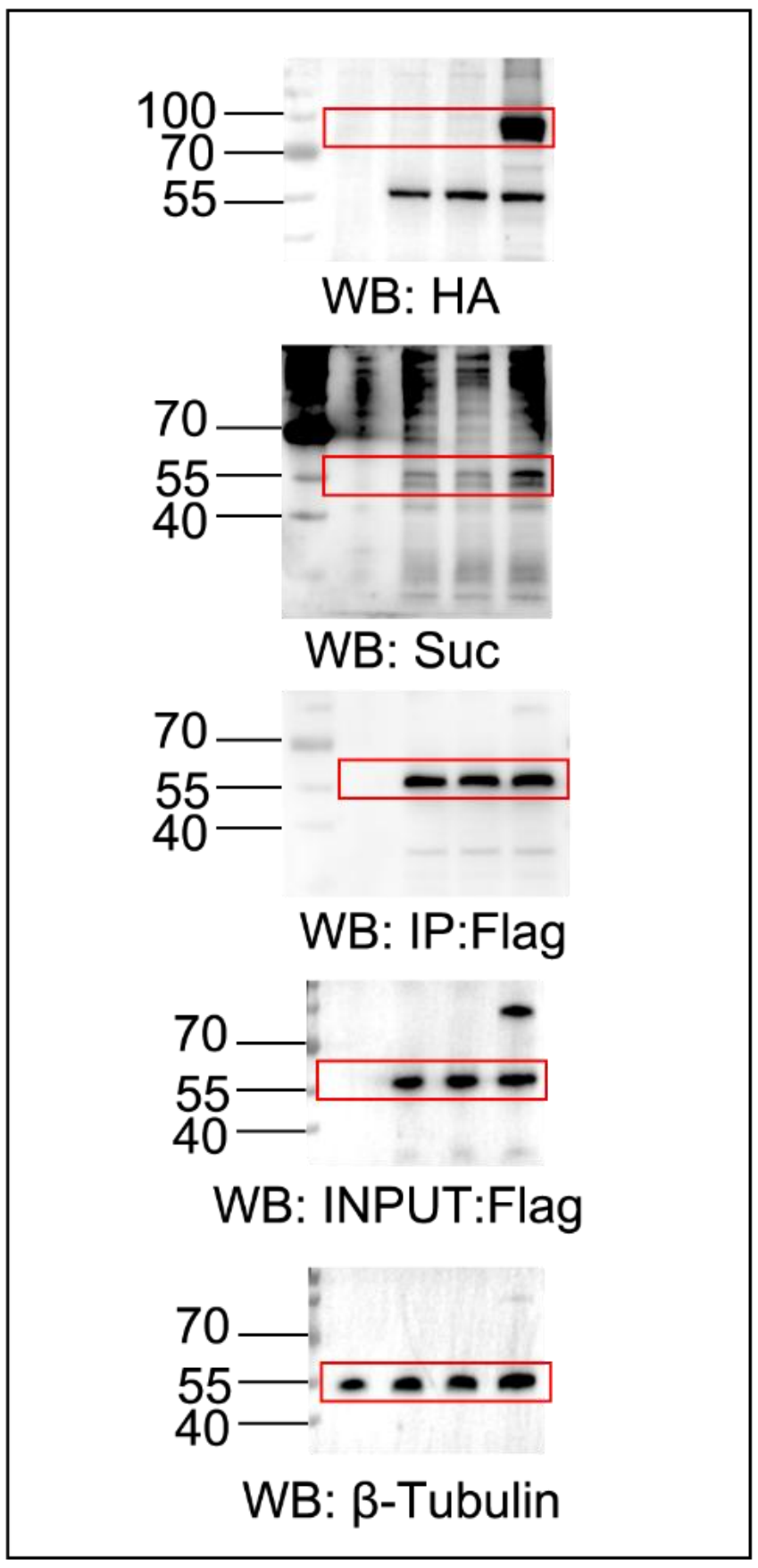

Figure 6K

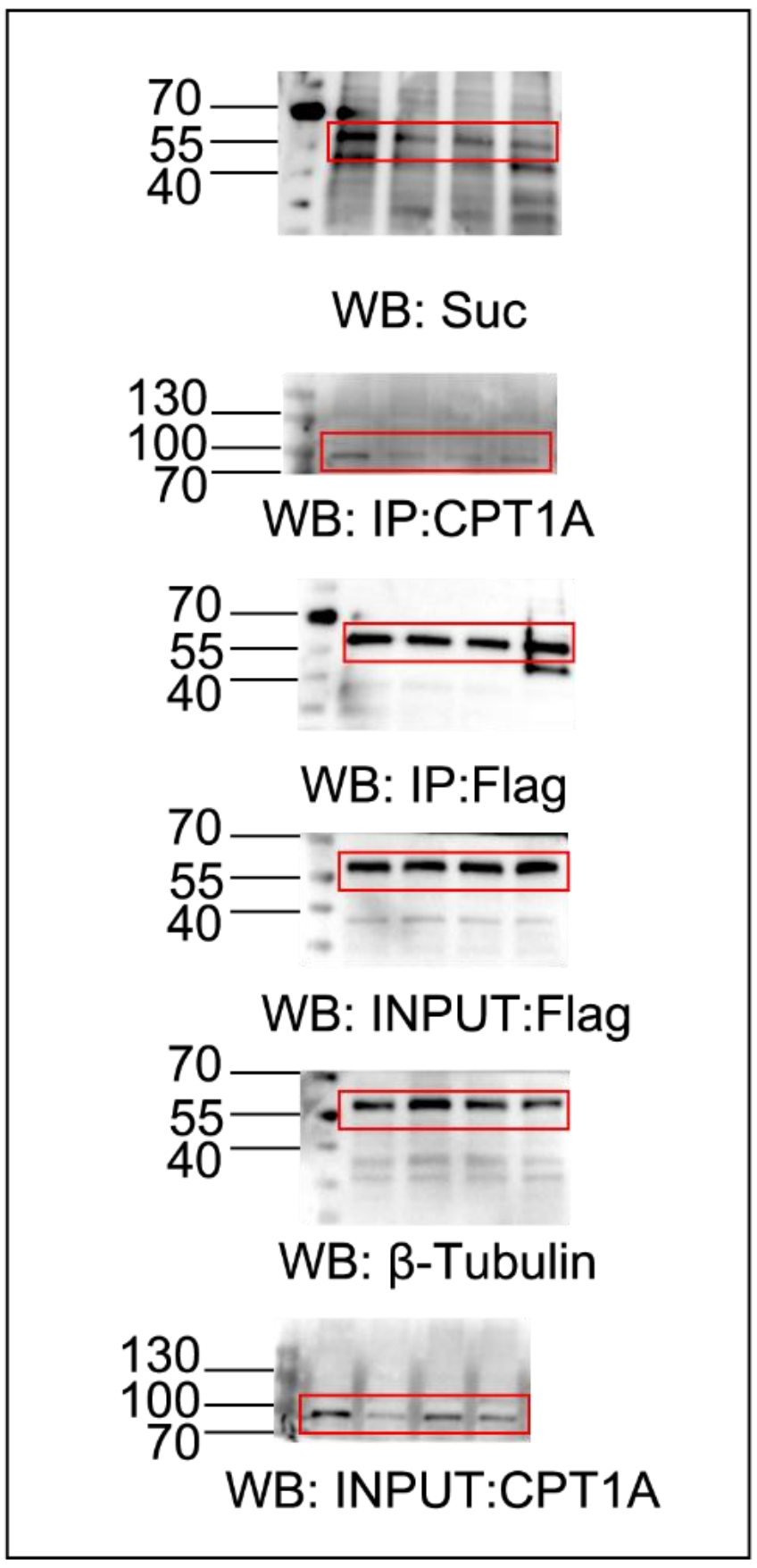

Figure 5I

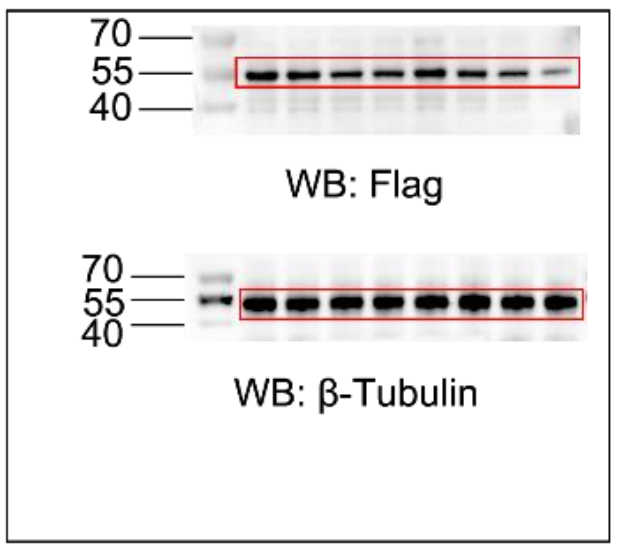

Figure 6M

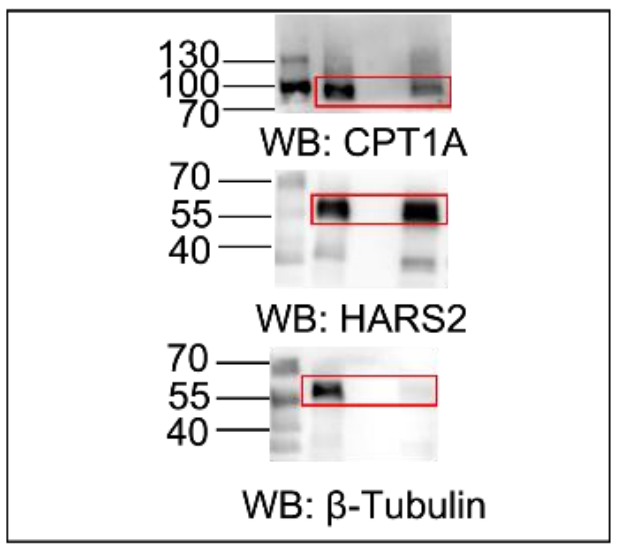

Figure 6N

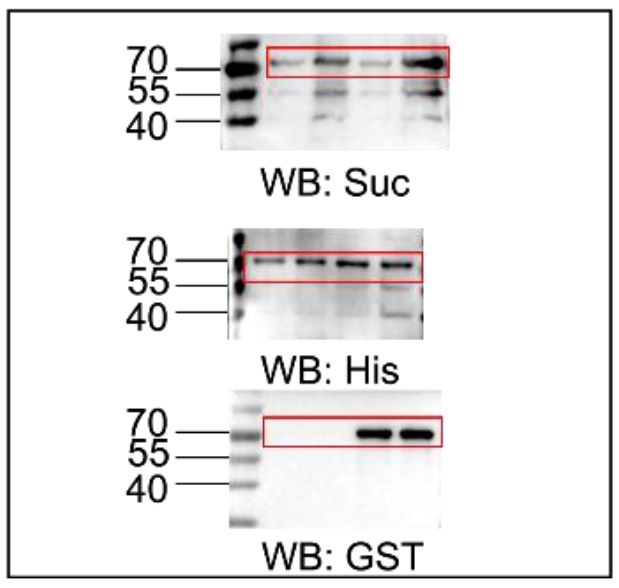

Figure 7A

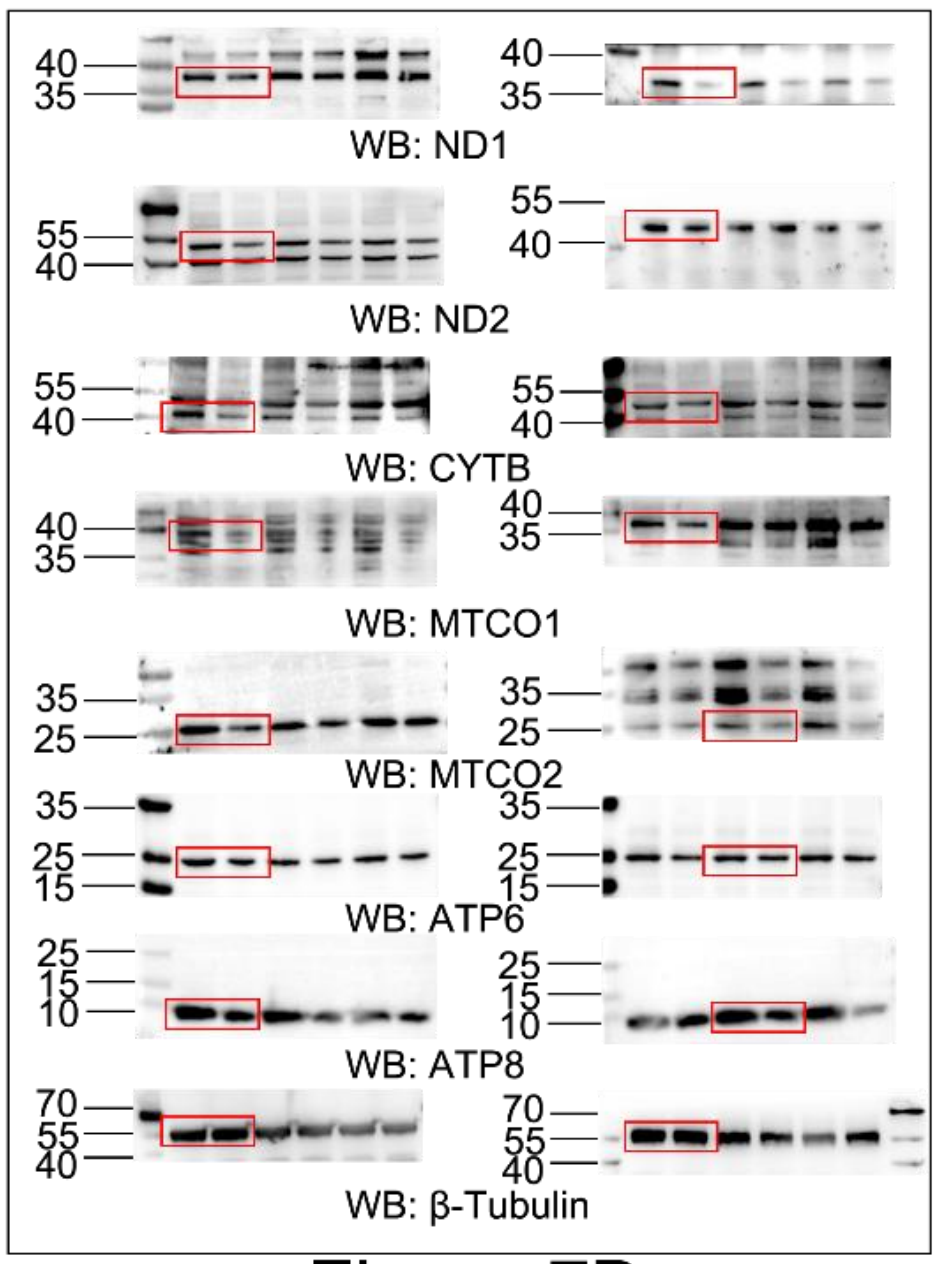

Figure 7E

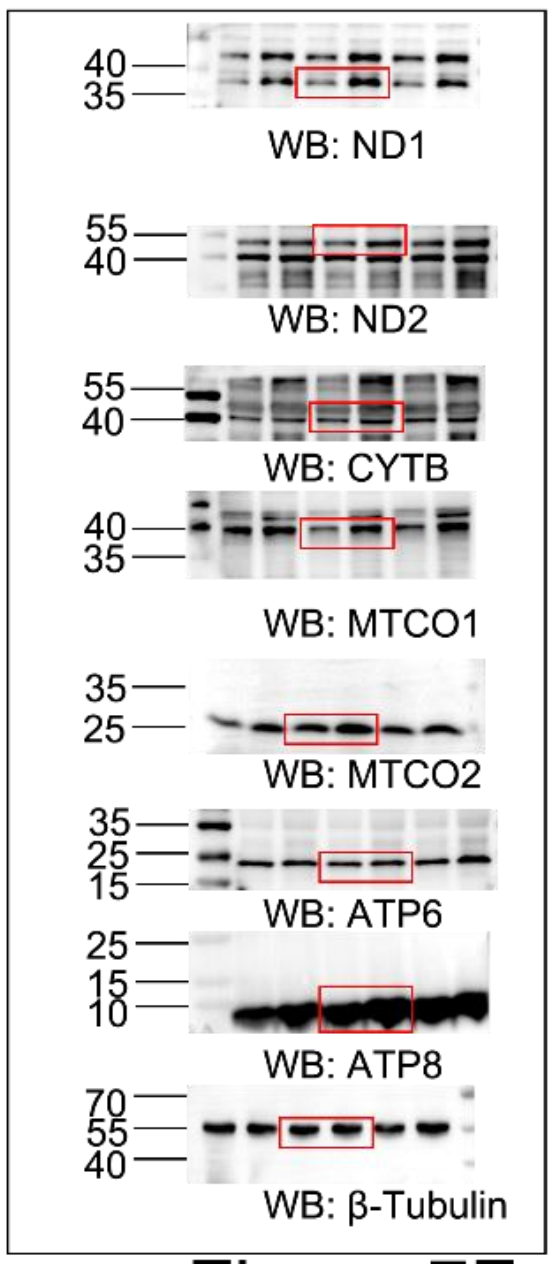

Figure 6L

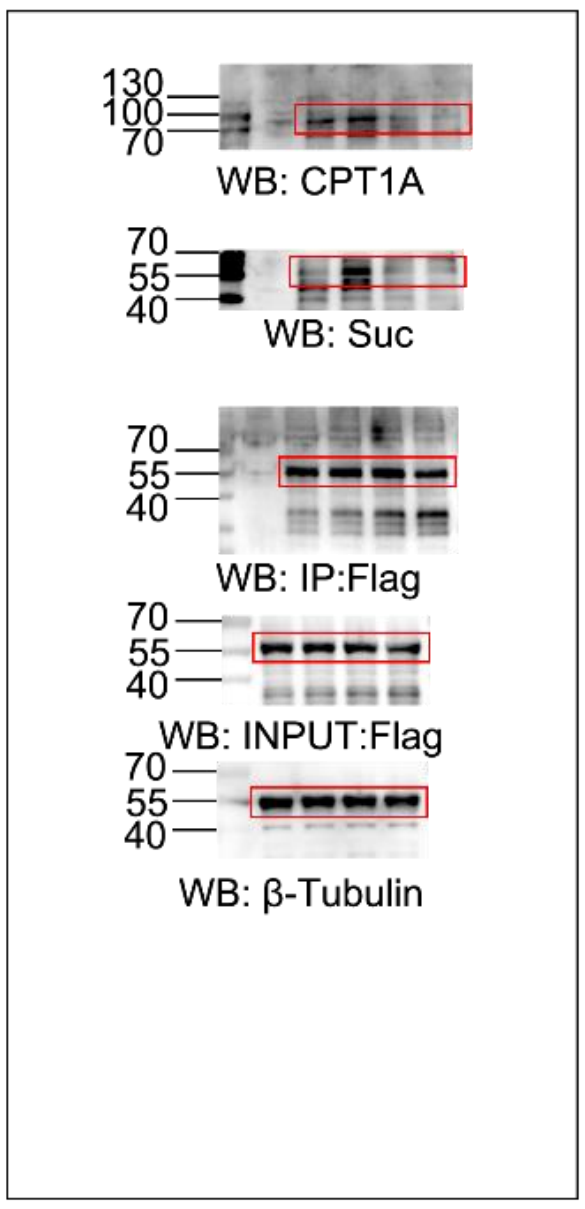

Figure 7B

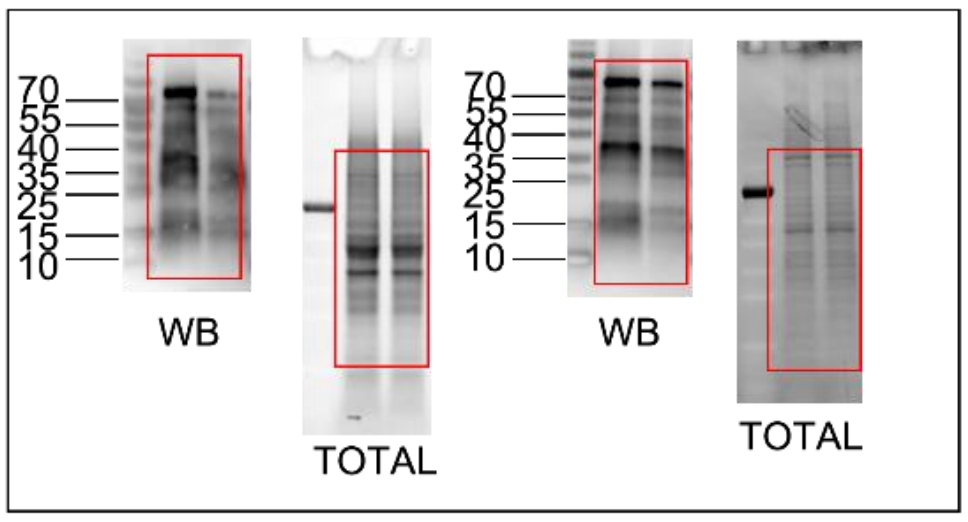

Figure 7F

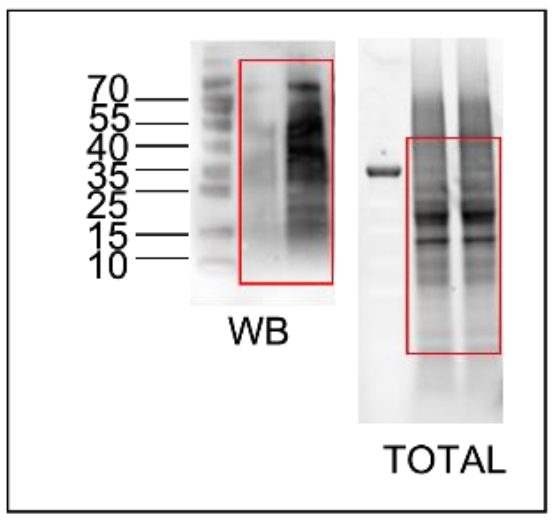

Figure 7I

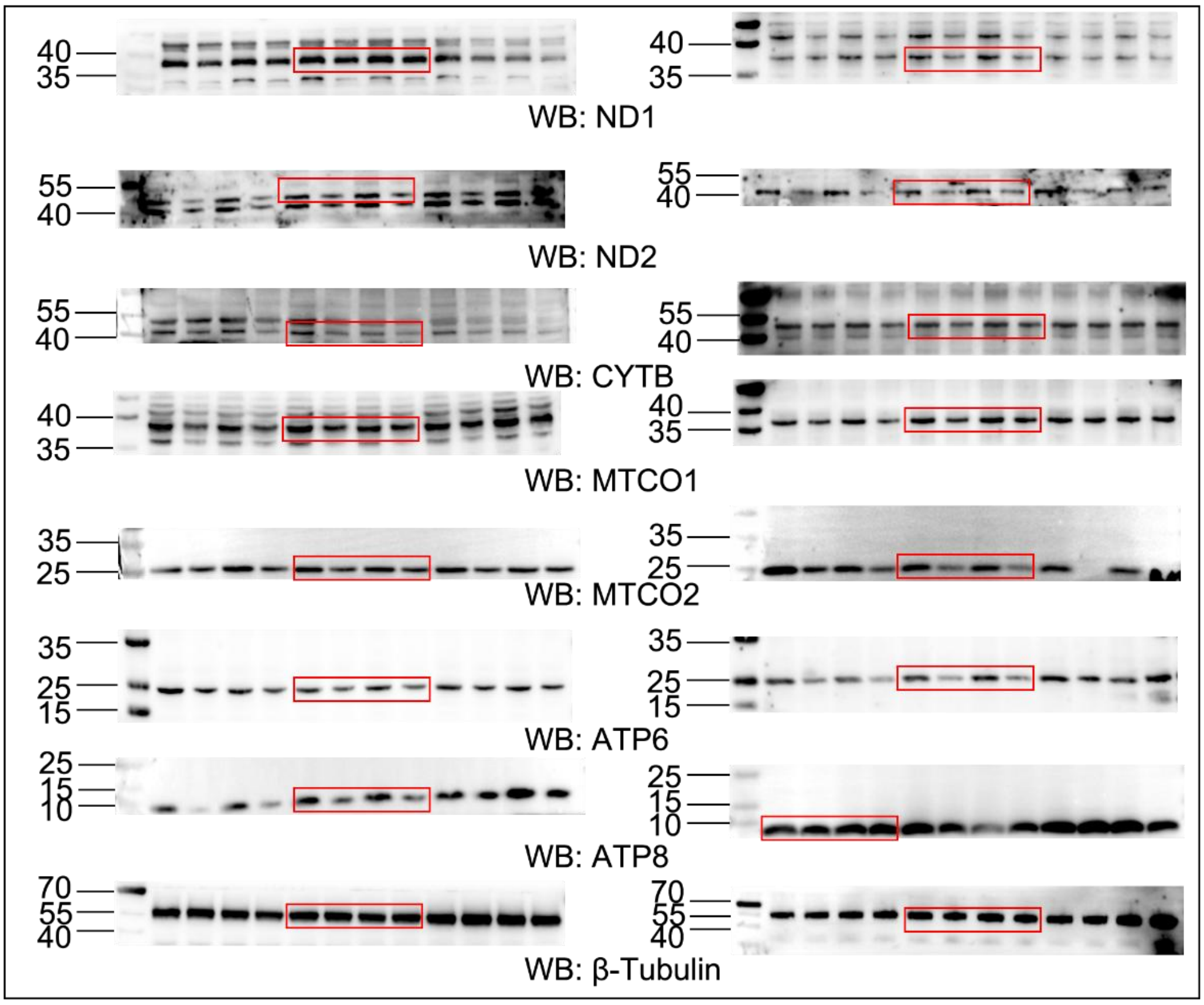

Figure 8I

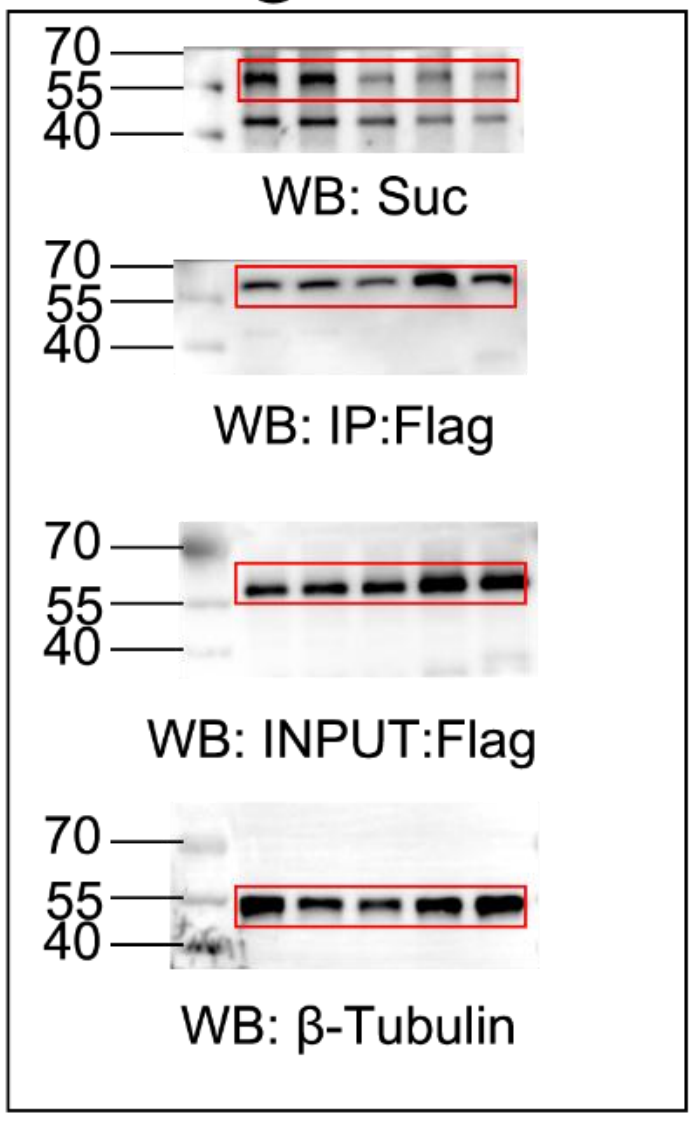

Supplement: Supplementary file 1 [file ADVS-13-e77042-s001.pdf]
