# Supplementary material for: A Novel tRNA Half mt‐5’‐tiRNA‐Tyr Promotes Colorectal Cancer Proliferation via Inducing HARS2 Succinylation
Source: Adv Sci (Weinh). 2026 Aug 21;13(57):e77042. doi: 10.1002/advs.77042 (PMC13496151; doi:10.1002/advs.77042)
Supplement: Supplementary file 2 [file ADVS-13-e77042-s002.docx]

**Fig. S1. Validation of mt‑5’‑tiRNA‑Tyr identity and sequencing features.**

**A** Read length distribution of reads mapped to the mt‑tRNA‑Tyr locus in pooled CRC tissues and adjacent tissues from the PANDORA‑seq data. **B** Top exact fragments mapped to the mt‑tRNA‑Tyr locus. The exact 31 nt mt‑5’‑tiRNA‑Tyr fragment sequence was GGTAAAATGGCTGAGTGAAGCATTGGACTGT. **C** Quantification of all 31 nt reads and exact mt‑5’‑tiRNA‑Tyr reads among mt‑tRNA‑Tyr locus reads. **D** Abundance of the exact 31 nt mt‑5’‑tiRNA‑Tyr fragment expressed as reads per million total tsRNA reads. **E** Agarose gel electrophoresis showing a single band of the expected size for the qRT‑PCR product of mt‑5’‑tiRNA‑Tyr. F Sanger sequencing confirmed that the qRT‑PCR product contained the mt‑5’‑tiRNA‑Tyr sequence.

**Fig. S2. mt-5’-tiRNA-Tyr is upregulated via HIF-1α/ANG axis**

**A** HIF-1α was detected to be overexpressed under the hypoxic environment in CRC cells using western blotting. **B** ANG was detected to be overexpressed in response to prolonged hypoxia time, as determined by western blotting. **C** mt-5’-tiRNA-Tyr was detected to be overexpressed in response to prolonged hypoxia time using qRT-PCR (*n* = 3). *****P* < 0.0001. **D** The knockdown of ANG significantly reduced the level of ANG. **E** The knockdown of ANG significantly reduced the level of mt-5’-tiRNA-Tyr (*n* = 3). *****P* < 0.0001, ****P* < 0.001. **F,G** The luciferase reporter gene assay was performed to validate the HIF-1α binding site on the ANG promoter (*n* = 3). *****P* < 0.0001, ****P* < 0.001, ns, not significant (*P* > 0.05). All values are means ± SD, and significance was determined by the one-way ANOVA followed by Tukey’s test (C,E) or the two-tailed Student’s *t*-test (G).

**Fig. S3. Enrichment analysis of transcriptome sequencing**

**A** PCA analysis of the mt-5’-tiRNA-Tyr inhibitor and the negative control. **B, C** KEGG and GO analysis of the transcriptome sequencing.

**Fig. S4. mt-5’-tiRNA-Tyr increases HARS2 protein stability.**

**A** The expression of HARS2 in TCGA dataset. ****P* < 0.001. **B** The expression of HARS2 in CRC tissues and adjacent tissues by IHC. Scale bar, up: 100 μm, down: 25 μm. **C** The expression of HARS2 was not affected by mt-5’-tiRNA-Tyr inhibitor or mimics by qRT-PCR (*n* = 3). **D** HARS2 staining of tumors in the mt-5’-tiRNA-Tyr down group is downregulated compared with the NC group. Scale bar, up: 20 μm. **E** The expression of HARS2 was detected by western blotting after transfection with mt-5’-tiRNA-Tyr mimics or inhibitor. ***P* < 0.01, **P* < 0.05. **F** The expression of HARS2 is higher in CRC cells than in NCM460 by qRT-PCR (*n* = 3). ***P* < 0.01, **P* < 0.05. **G** After overexpression of HARS2, the protein expression of HARS2 was verified in SW480 cells. **H** After knockdown of HARS2, the protein expression of HARS2 was verified in SW480 cells. **I** SW480 cells were transfected with the indicated plasmids and then treated with CHX (100 μg/mL) for 0, 4, 8, and 12 hours. The degradation rate of HARS2 was detected by western blotting. All values are means ± SD, and significance was determined by the two-tailed Student’s t-test (A,C,E) or one-way ANOVA followed by Tukey’s test (F).

**Fig. S5. Other post-translational modifications of HARS2**

**A** Co-IP and western blotting were used to detect the methylation level of HARS2. **B** Co-IP and western blotting were used to detect the acetylation level of HARS2. **C** Co-IP and western blotting were used to detect the lactylation level of HARS2.

**Fig. S6. Validation of the HPG-based nascent mitochondrial protein synthesis assay under emetine treatment.**

Representative HPG-labeled nascent protein signals in SW480 and RKO cells with or without emetine treatment. Total protein staining was used as a loading control.

**Fig. S7. The correlation between mt-5'-tiRNA-Tyr and common clinical tumor markers**

**A-E** Correlation analysis between plasma mt-5’-tiRNA-Tyr expression and CEA (A), CA19-9 (B), CA72-4 (C), CA24-2 (D), and CA50 (E) levels in CRC patients. Correlations were evaluated using Spearman correlation analysis.

**Fig. S8. mt-5’-tiRNA-Tyr serves as a plasma biomarker for CRC.**

**A** qRT-PCR analysis of plasma mt-5’-tiRNA-Tyr expression in CRC patients (*n* = 104), patients with intestinal polyps (*n* = 50), and healthy donors (*n* = 98). **B,C** Plasma mt-5’-tiRNA-Tyr expression in stage III-IV CRC patients (B) and stage I-II CRC patients (C) compared with healthy donors. **D,E** Plasma mt-5’-tiRNA-Tyr expression was higher in stage III-IV CRC than in stage I-II CRC (D) and higher in T3/T4 tumors than in T1/T2 tumors (E). **F** Paired analysis of plasma mt-5’-tiRNA-Tyr expression before and after surgery. ROC analysis of mt-5’-tiRNA-Tyr, CEA, CA19-9, CA72-4, CA50, CA24-2, and the combined panel for distinguishing CRC patients from healthy donors in the training cohort (G) and internal validation cohort (H). **I** ROC analysis for distinguishing early-stage CRC patients from healthy donors. **J** ROC analysis for distinguishing CRC patients from intestinal polyp patients. All values are median with interquartile range unless otherwise indicated, and significance was determined by the Kruskal-Wallis test followed by Dunn’s multiple-comparison test (A), two-tailed Mann-Whitney U test (B-E), or paired two-tailed Wilcoxon test (F).

**Table S1. Primers used in this study.**

| **Name** | **Sequences (5’-3’)** |
| --- | --- |
| mt-5’-tiRNA-Tyr RT | GTCGTATCCAGTGCAGGGTCCGAGGTATTCGCACTGGATACGACACAGTC |
| mt-5’-tiRNA-Tyr F | AAAATGGCTGAGTGAAGCATTG |
| mt-5’-tiRNA-Tyr R | AGTGCAGGGTCCGAGGTATT |
| U6 RT | CGCTTCACGAATTTGCGTGTCAT |
| U6 F | GCTTCGGCAGCACATATACTAAAAT |
| U6 R | CGCTTCACGAATTTGCGTGTCAT |
| HARS2 F | TTCCGTGCCATCTGCTCCTC |
| HARS2 R | CCAATTCGATCAGCCACCTCAG |
| β-actin F | TCAAGATCATTGCTCCTCCTGAG |
| β-actin R | ACATCTGCTGGAAGGTGGACA |

**Table S2. The sequences of inhibitor, mimics, siRNAs, lentiviral vectors used in this study.**

| **Oligo name** | **Sequences (5’-3’)** |
| --- | --- |
| mt-5’-tiRNA-Tyr mimics sense | GGUAAAAUGGCUGAGUGAAGCAUUGGACUGU |
| mt-5’-tiRNA-Tyr mimics antisense | AGUCCAAUGCUUCACUCAGCCAUUUUACCUU |
| mt-5’-tiRNA-Tyr inhibitor | ACAGUCCAAUGCUUCACUCAGCCAUUUUACC |
| siANG-1 sense | CCAGCACUAUGAUGCCAAATT |
| siANG-1 antisense | UUUGGCAUCAUAGUGCUGGTT |
| siANG-2 sense | GCAACAAGCGCAGCAUCAATT |
| siANG-2 antisense | UUGAUGCUGCGCUUGUUGCTT |
| siANG-3 sense | GGUCACCACUUGCAAGCUATT |
| siANG-3 antisense | UAGCUUGCAAGUGGUGACCTT |
| siCPT1A-1 sense | GCGUGAUGACAACGAUGUATT |
| siCPT1A-1 antisense | UACAUCGUUGUCAUCACGCTT |
| siCPT1A-2 sense | GGUGGAUGUUCACUGAGCATT |
| siCPT1A-2 antisense | UGCUCAGUGAACAUCCACCTT |
| siCPT1A-3 sense | GGAUGGGUAUGGUCAAGAUTT |
| siCPT1A-3 antisense | AUCUUGACCAUACCCAUCCTT |
| mt-5’-tiRNA-Tyr down | ACAGTCCAATGCTTCACTCGACATTTTACCCTTCACAGTCCAATGCTTCACTCGACATTTTACCCTTCACAGTCCAATGCTTCACTCGACATTTTACCCTTCACAGTCCAATGCTTCACTCGACATTTTACCCTTCACAGTCCAATGCTTCACTCGACATTTTACCCTTCACAGTCCAATGCTTCACTCGACATTTTACC |

**Table S3. Antibodies used in this study.**

| Antibodies | SOURCE | IDENTIFIER |
| --- | --- | --- |
| ANG I antibody (C-1) | santa cruz | sc-74528 |
| HARS2 Polyclonal antibody | Proteintech | 11301-1-AP |
| ND1 Polyclonal antibody | Proteintech | 19703-1-AP |
| CYTB Polyclonal antibody | Proteintech | 55090-1-AP |
| ATP6 Rabbit mAb (A23150) | Abclonal | A23150 |
| MTCO2 Polyclonal antibody | Proteintech | 55070-1-AP |
| ATP8 Rabbit mAb (A27711) | Abclonal | A27711 |
| ND2 Polyclonal antibody | Proteintech | 19704-1-AP |
| MTCO1 Rabbit pAb | Abclonal | A17889 |
| Anti-L-Lactyl Lysine Rabbit pAb | PTM BIO | PTM-1401 |
| Anti-Succinyllysine Mouse mAb | PTM BIO | PTM-419 |
| Pan Acetylation Monoclonal antibody | Proteintech | 66289-1-Ig |
| Methyl-Lysine Antibody | NOVUS | NB600-824 |
| CSRP2BP Rabbit pAb (A7380) | ABclonal | A7380 |
| DDDDK-Tag Rabbit mAb (AE092) | ABclonal | AE092 |
| HRP-conjugated Rabbit anti HA-Tag mAb | ABclonal | AE123 |
| CPT1A Rabbit pAb (A5307) | ABclonal | A5307 |

**Table S4. Diagnostic performance of mt-5'-tiRNA-Tyr, conventional tumor markers, and the combined panel in the training cohort.**

| **Model** | **AUC** | **95% CI** | **Cutoff** | **Sensitivity** | **Specificity** | **Accuracy** | **DeLong comparison** |
| --- | --- | --- | --- | --- | --- | --- | --- |
| mt-5'-tiRNA-Tyr | 0.912 | 0.858-0.966 | 3.452 | 0.836 | 0.956 | 0.894 | reference |
| CEA | 0.725 | 0.641-0.808 | 2.890 | 0.589 | 0.779 | 0.681 | vs mt-5'-tiRNA-Tyr, P < 0.001 |
| CA19-9 | 0.618 | 0.523-0.712 | 16.350 | 0.384 | 0.941 | 0.652 | vs mt-5'-tiRNA-Tyr, P < 0.001 |
| CA72-4 | 0.592 | 0.498-0.687 | 1.820 | 0.562 | 0.544 | 0.553 | vs mt-5'-tiRNA-Tyr, P < 0.001 |
| CA50 | 0.795 | 0.721-0.868 | 4.650 | 0.808 | 0.676 | 0.745 | vs mt-5'-tiRNA-Tyr, P = 0.008 |
| CA24-2 | 0.766 | 0.688-0.843 | 4.950 | 0.644 | 0.765 | 0.702 | vs mt-5'-tiRNA-Tyr, P = 0.003 |
| Combined panel | 0.937 | 0.896-0.977 | 0.407 | 0.849 | 0.912 | 0.879 | vs mt-5'-tiRNA-Tyr, *P* = 0.361 |

**Table S5. Diagnostic performance of mt-5'-tiRNA-Tyr, conventional tumor markers, and the combined panel in the internal validation cohort.**

| **Model** | **AUC** | **95% CI** | **Cutoff** | **Sensitivity** | **Specificity** | **Accuracy** | **DeLong comparison** |
| --- | --- | --- | --- | --- | --- | --- | --- |
| mt-5'-tiRNA-Tyr | 0.889 | 0.794-0.984 | 3.452 | 0.710 | 0.933 | 0.820 | reference |
| CEA | 0.699 | 0.564-0.834 | 2.890 | 0.645 | 0.533 | 0.590 | vs mt-5'-tiRNA-Tyr, *P* = 0.009 |
| CA19-9 | 0.582 | 0.430-0.733 | 16.350 | 0.355 | 0.900 | 0.623 | vs mt-5'-tiRNA-Tyr, *P* = 0.003 |
| CA72-4 | 0.510 | 0.360-0.660 | 1.820 | 0.484 | 0.533 | 0.508 | vs mt-5'-tiRNA-Tyr, *P* < 0.001 |
| CA50 | 0.761 | 0.629-0.894 | 4.650 | 0.710 | 0.733 | 0.721 | vs mt-5'-tiRNA-Tyr, *P* = 0.154 |
| CA24-2 | 0.642 | 0.496-0.788 | 4.950 | 0.484 | 0.700 | 0.590 | vs mt-5'-tiRNA-Tyr, *P* = 0.007 |
| Combined panel | 0.941 | 0.887-0.995 | 0.407 | 0.839 | 0.933 | 0.885 | vs mt-5'-tiRNA-Tyr, *P* = 0.274 |
